# The symbiotic axis between the acidic tumor microenvironment and cancer stem cells: a driver of malignancy and therapeutic resistance

**DOI:** 10.3389/fonc.2026.1804379

**Published:** 2026-03-26

**Authors:** Zhengyu Wang, Hanghang Yuan, Mei Liu, Jupeng Yuan, Dawei Chen

**Affiliations:** 1 School of Clinical Medicine, Shandong Second Medical University, Weifang, Shandong, China; 2 Department of Shandong Provincial Key Laboratory of Precision Oncology, Shandong Cancer Hospital and Institute, Shandong First Medical University and Shandong Academy of Medical Sciences, Jinan, Shandong, China; 3 Cancer Center, Shandong University, Jinan, Shandong, China; 4 The Affiliated Cancer Hospital of Xinjiang Medical University, Xinjiang Key Laboratory of Oncology, Urumqi, Xinjiang, China

**Keywords:** acidic tumor microenvironment, cancer stem cells, histone lactylation, metabolic remodeling, stromal reprogramming, symbiotic axis

## Abstract

The physicochemical conditions of tumors—such as acidosis, stiffness, and hypoxia—actively drive cancer progression yet have been insufficiently incorporated into the current hallmarks of cancer model. This review focuses on one such dominant condition, the acidic tumor microenvironment (ATME), and its dynamic interplay with cancer stem cells (CSCs), proposing their cooperative interaction as a central driver of tumor aggressiveness. We present evidence that acidosis, driven by glycolytic metabolism and proton extrusion, epigenetically reprograms tumor cells toward a stem-like state through mechanisms including histone lactylation. In turn, CSCs reinforce acidity through metabolic remodeling and stromal reprogramming, forming a self-sustaining feedback loop. This ATME-CSC axis underpins key cancer hallmarks: uncontrolled self-renewal, metabolic adaptability, immune evasion, and metastasis. Disrupting this axis requires multi-target strategies that concurrently neutralize niche acidity, target CSC metabolism, and reset associated epigenetic programs. Future advances will depend on spatial mapping of this axis *in vivo*, development of microenvironment-responsive agents, and functional validation using patient-derived organoids. Targeting the symbiotic interface between tumor acidity and stemness offers a transformative pathway to durable therapeutic control.

## 1 Introduction

Therapeutic resistance, relapse, and metastasis continue to be the leading causes of cancer-related mortality. These clinical challenges are closely associated with a distinct subpopulation of cells within tumors—cancer stem cells (CSCs). CSCs are characterized by their remarkable self-renewal capacity, ability to differentiate, and, most importantly, their phenotypic plasticity. This adaptability enables CSCs to persist, regenerate tumors, and drive tumor progression following therapy (1). Importantly, the CSC state is not static but is instead dynamically regulated by extrinsic signals from the tumor microenvironment (TME) (2).

Among the various physicochemical stressors in the TME, extracellular acidosis stands out as a dominant and instructive force. The acidic tumor microenvironment (ATME), primarily driven by glycolytic metabolism and characterized by a pH as low as 6.5, is much more than a passive byproduct of dysregulated metabolism (3). We and others propose that the ATME and CSCs do not merely coexist; instead, they engage in a symbiotic, self-reinforcing cycle—the ATME-CSC axis (4).

Within this axis, the ATME actively reprograms the CSC state through metabolic reprogramming and epigenetic remodeling. For example, adaptation to acidic conditions can directly upregulate stemness markers such as SOX2 and enhance tumorigenicity (5). In turn, CSCs actively shape and amplify their acidic niche by overexpressing proton exporters like MCTs and CA IX, thus creating a feed-forward loop that accelerates malignancy (6).

The discovery of histone lactylation has provided a direct molecular link, revealing how lactate—a key metabolite in the ATME—can directly modify chromatin and promote the expression of stemness genes (7). This illustrates how the ATME-CSC axis functions through epigenetic reprogramming. Genome-wide studies show that lactylation marks, such as H3K18la, are enriched at the promoters of pluripotency genes (8), and this mechanism drives CSC properties in glioblastoma and liver cancer. At the same time, CSCs within this niche exhibit remarkable metabolic plasticity, alternately utilizing glycolysis or oxidative phosphorylation to survive stress, which contributes to the conflicting findings in the literature and poses a major challenge for therapeutic intervention (9).

The ATME-CSC axis underlies the acquisition of key cancer hallmarks: it drives uncontrolled, therapy-resistant proliferation, orchestrates metastatic dissemination, and systematically remodels the immune landscape into an immunosuppressive fortress. As such, the persistence of this axis serves as a fundamental biological basis for treatment failure.

This review synthesizes recent advances to establish a unified framework for understanding the ATME-CSC axis. We first explore how acidity shapes and sustains the CSC state. We then examine how this axis drives the malignant program, promoting proliferation, metastasis, and immune evasion. Finally, moving beyond single-target therapies, we propose a multi-layered therapeutic paradigm aimed at disrupting this resilient cycle by simultaneously neutralizing the niche, targeting CSC metabolic plasticity, and erasing the malignant epigenetic program, while also highlighting future directions for personalized interventions.

## 2 The dynamic construction and multifaceted impact of the ATME

### 2.1 Metabolic reprogramming as the driver of tumor acidosis

The formation of an acidic extracellular milieu is primarily a thermodynamic result of the metabolic reprogramming in cancer cells. This reprogramming collectively enhances the net production and export of protons (H^+^), surpassing the local buffering capacity and causing a prolonged decrease in extracellular pH (pHe). This process is regulated at various levels of metabolism.

#### 2.1.1 Enhanced glycolytic flux: a primary source of protons

A hallmark of many cancers is the increased dependence on glycolysis, even in the presence of oxygen (the Warburg effect). This shift is driven by the upregulation of key glycolytic enzymes. For example, hexokinase 2 (HK2) facilitates glucose uptake and commits cells to glycolysis (10). Notably, the terminal step—the conversion of pyruvate to lactate, catalyzed by lactate dehydrogenase A (LDHA)—regenerates NAD^+^ to sustain glycolysis but also produces lactic acid (lactate^−^ and H^+^ in equilibrium). The concurrent export of lactate^−^ and H^+^ via monocarboxylate transporters (MCTs, particularly MCT4) directly acidifies the extracellular space (11). This connection is further illustrated by studies showing that oncogenic signals can upregulate pyruvate kinase M2 (PKM2), accelerating glycolytic flux and lactate/H^+^ efflux, thereby fostering an invasive microenvironment (12).

#### 2.1.2 Rewired mitochondrial metabolism: reducing proton-consuming pathways

Concomitant with the upregulation of glycolysis is a reprogramming of mitochondrial function. While mitochondria are not universally inactive, in many tumor contexts, they exhibit metabolic adaptations that support extracellular acidification. Inhibition or downregulation of oxidative phosphorylation (OXPHOS) diminishes the primary intracellular pathway for consuming pyruvate and protons through the electron transport chain (13). This metabolic shift can be further exacerbated by the acidic environment itself. For instance, lactate accumulation and low pH have been shown to suppress mitochondrial biogenesis and OXPHOS activity, thereby reinforcing glycolytic dependency (14). Such mitochondrial rewiring ensures that glycolytically produced pyruvate is primarily diverted to lactate rather than being oxidized, thus sustaining the net proton export.

#### 2.1.3 Integrated regulation: the role of hypoxia-inducible factors

The metabolic shift toward a proton-producing phenotype is often master-regulated by hypoxia-inducible factors (HIFs), which are stabilized not only by hypoxia but also by metabolites such as succinate and the acidic microenvironment itself. HIF-1α transactivates genes encoding glycolytic enzymes (e.g., HK2, PKM2, LDHA), glucose transporters (GLUT1), orchestrating a coherent program that maximizes glucose-derived lactate and H^+^ production and efflux (15). Furthermore, HIF-1α can suppress mitochondrial activity by inducing pyruvate dehydrogenase kinase (PDK), which inhibits the entry of pyruvate into the TCA cycle (16). This HIF-mediated coordination establishes a robust feed-forward loop that drives and sustains tumor acidosis.

#### 2.1.4 Beyond the Warburg paradigm: metabolic plasticity and context-dependence

It is important to note that metabolic reprogramming is both heterogeneous and plastic. Not all tumor cells or subpopulations (such as certain cancer stem cells) exhibit a purely glycolytic phenotype; some may rely on OXPHOS or dynamically switch between metabolic modes in response to microenvironmental cues (17, 18). This metabolic plasticity is not independent but is deeply entwined with the regulation of the acidic niche. Research indicates that acidosis itself can act as a selector and driver of specific metabolic phenotypes, creating a feed-forward loop that reinforces both the metabolic state and the acidic ecosystem (19). Despite this underlying heterogeneity, the net effect at the bulk tumor tissue level is consistently a substantial increase in glycolytic flux coupled with elevated proton export. This establishes the ATME not as an incidental byproduct, but as a common, ecosystem-level hallmark that actively shapes tumor evolution and therapeutic response (20).

### 2.2 The acidic niche: composition, maintenance, and cellular sensing

The ATME is characterized by a steady-state extracellular pH (pHe) typically ranging from 6.5 to 6.9, in contrast to the physiological pHe of approximately 7.4 in normal tissues (3). This proton-rich milieu is not a static condition but rather a dynamic equilibrium maintained by the coordinated action of tumor and stromal cells through specialized transport and buffering systems. Importantly, cells have developed sophisticated mechanisms to sense and respond to this extracellular acidosis.

#### 2.2.1 Proton export systems that acidify the extracellular space

As detailed in section 1.1, glycolysis generates equimolar lactate^−^ and H^+^. To prevent lethal intracellular acidification, tumor cells efficiently expel these protons. This efflux is largely driven by the aforementioned HIF-1α regulatory network, which upregulates monocarboxylate transporters (MCTs), primarily MCT1 and MCT4, to facilitate the electroneutral co-transport of lactate^−^ and H^+^ across the plasma membrane. While this maintains intracellular pH (pHi), it directly contributes to extracellular acidification (21). The expression and activity of MCT4, in particular, are often upregulated by hypoxia and acidosis, creating a feed-forward loop for proton export (15). Vacuolar-type H^+^-ATPase (V-ATPase) is another ATP-dependent proton pump that actively transports H^+^ from the cytoplasm to the extracellular space, independent of lactate flux. Overexpression of V-ATPase in tumor cells is a major contributor to the ATME and is linked to invasion, drug resistance, and immune evasion (22).

#### 2.2.2 Carbonic anhydrase IX: a key pH-regulatory enzyme

Induced primarily by hypoxia, CA IX is a tumor-associated enzyme that is anchored to the extracellular membrane. It catalyzes the reversible hydration of CO_2_ to form carbonic acid (H_2_CO_3_), which then rapidly dissociates into H^+^ and bicarbonate (HCO_3_^−^) (23). This reaction plays a dual, paradoxical role in the ATME (1): the generated H^+^ is released locally, contributing to the acidification of the extracellular space; (2) the bicarbonate ion can be imported into the cell, often via anion exchangers, to counteract intracellular acidosis, thereby promoting cell survival in a harsh microenvironment (24). In this way, CA IX activity simultaneously acidifies the extracellular space while alkalinizing the interior of tumor cells.

#### 2.2.3 Cellular sensors of extracellular acidosis: ASICs and GPCRs

To adapt and respond to low extracellular pH (pHe), cells express specific proton-sensing receptors on their surface, such as Acid-Sensing Ion Channels (ASICs). These channels open in response to a drop in pHe, allowing an influx of Na^+^ and Ca²^+^. This ionic flux can activate downstream signaling pathways that promote proliferation, migration, and survival. For instance, the expression of Acid-Sensing Ion Channel 1a (ASIC1a) is upregulated in HepG2 cells in response to the acidic microenvironment, enhancing cellular migration and invasion by modulating autophagic flux. Inhibition of ASIC1a reduces autophagic activity, thereby attenuating the migratory and invasive capacities of hepatocellular carcinoma (HCC) cells (25). Proton-Sensing G Protein-Coupled Receptors (GPCRs), such as GPR4, are also activated by extracellular protons (26). Their activation triggers diverse intracellular signaling cascades that regulate cell adhesion, cytokine production, and survival, further influencing tumor progression and shaping the immune landscape (27).

In summary, the ATME is actively established and maintained by proton-exporting transporters (MCTs, V-ATPase) and enzymes (CA IX), while its biological effects are mediated through specific proton-sensing receptors (ASICs, GPCRs). This integrated system enables tumors to thrive in acidic conditions that are detrimental to normal tissue function.

### 2.3 The acidic microenvironment as an active signaling hub: multifaceted biological effects

Beyond being a mere metabolic byproduct, the ATME serves as a potent signaling entity that actively reshapes the tumor ecosystem. The combined effects of low pH and elevated lactate orchestrate a series of phenotypic changes in tumor cells, immune infiltrates, and stromal components, collectively driving invasion, immune evasion, and metastatic progression.

#### 2.3.1 Reshaping tumor cells and the stroma: facilitating invasion and metastasis

The ATME directly enhances the invasive capacity of tumor cells. Low pH upregulates the expression and activity of matrix metalloproteinases (MMPs), enzymes that degrade the extracellular matrix (ECM), thereby removing physical barriers to cell migration (28). Concurrently, acidosis induces epithelial-mesenchymal transition (EMT), a process that reduces cell-cell adhesion and increases motility, often through the activation of pathways such as TGF-β signaling (29, 30). This effect is further amplified by acidosis-altered intercellular communication. For instance, tumor-derived exosomes secreted under acidic conditions carry a distinct cargo of pro-invasive miRNAs, proteins, and lipids. These exosomes can remodel the pre-metastatic niche in distant organs, enhancing endothelial permeability and creating a supportive environment for circulating tumor cells to colonize (31).

#### 2.3.2 Reprogramming the immune landscape: establishing an immunosuppressive barrier

A defining feature of the ATME is its profound capacity to suppress antitumor immunity. Extracellular acidosis and lactate directly impair the function of key immune effector cells. Research shows that the acidic condition of pH 6.0-6.5 can directly induce a dysfunctional state in tumor-infiltrating CD8^+^ T cells. This state is characterized by a significant decrease in the secretion of cytokines (such as IFN-γ and TNF) and impaired cytotoxic killing function (32). Furthermore, acidosis-driven lactate accumulation can also inhibit CD8^+^ T cell function by activating the MondoA-TXNIP (33). Lactate, acting through receptors such as GPR81 on immune cells, transmits direct inhibitory signals that dampen immune activation (34). While traditionally classified into pro-inflammatory (M1) and immunosuppressive (M2) subsets, it is now recognized that macrophages exist along a continuous spectrum of activation states, dynamically adapting to microenvironmental cues (35). Moreover, the ATME drives macrophage reprogramming toward a pro-tumorigenic, immunosuppressive state along their activation spectrum. These tumor-associated macrophages (TAMs) secrete immunosuppressive cytokines like IL-10 and TGF-β, further paralyzing T cell responses and promoting tissue remodeling (36). Recent studies suggest that lactate can induce protein lactylation in immune and stromal cells, adding a novel epigenetic layer that stabilizes this immunosuppressive state (37).

#### 2.3.3 Activating and educating stromal cells: building a supportive niche

The ATME dynamically interacts with and activates cancer-associated fibroblasts (CAFs). Lactate exported from tumor cells is taken up by neighboring CAFs, inducing metabolic and transcriptional reprogramming that enhances their pro-tumor functions (38). Activated CAFs, in turn, secrete a variety of factors that fuel tumor progression. They produce ECM components (e.g., collagen) to stiffen the stroma, growth factors (e.g., VEGF, FGF2) to stimulate angiogenesis, and chemokines (e.g., CXCL13) that recruit immunosuppressive cell types such as regulatory B cells (Bregs) (39, 40). This creates a vicious cycle in which the ATME-educated stroma provides both structural support and chemical signals that reinforce tumor survival, growth, and immune evasion.

**Figure 1:**
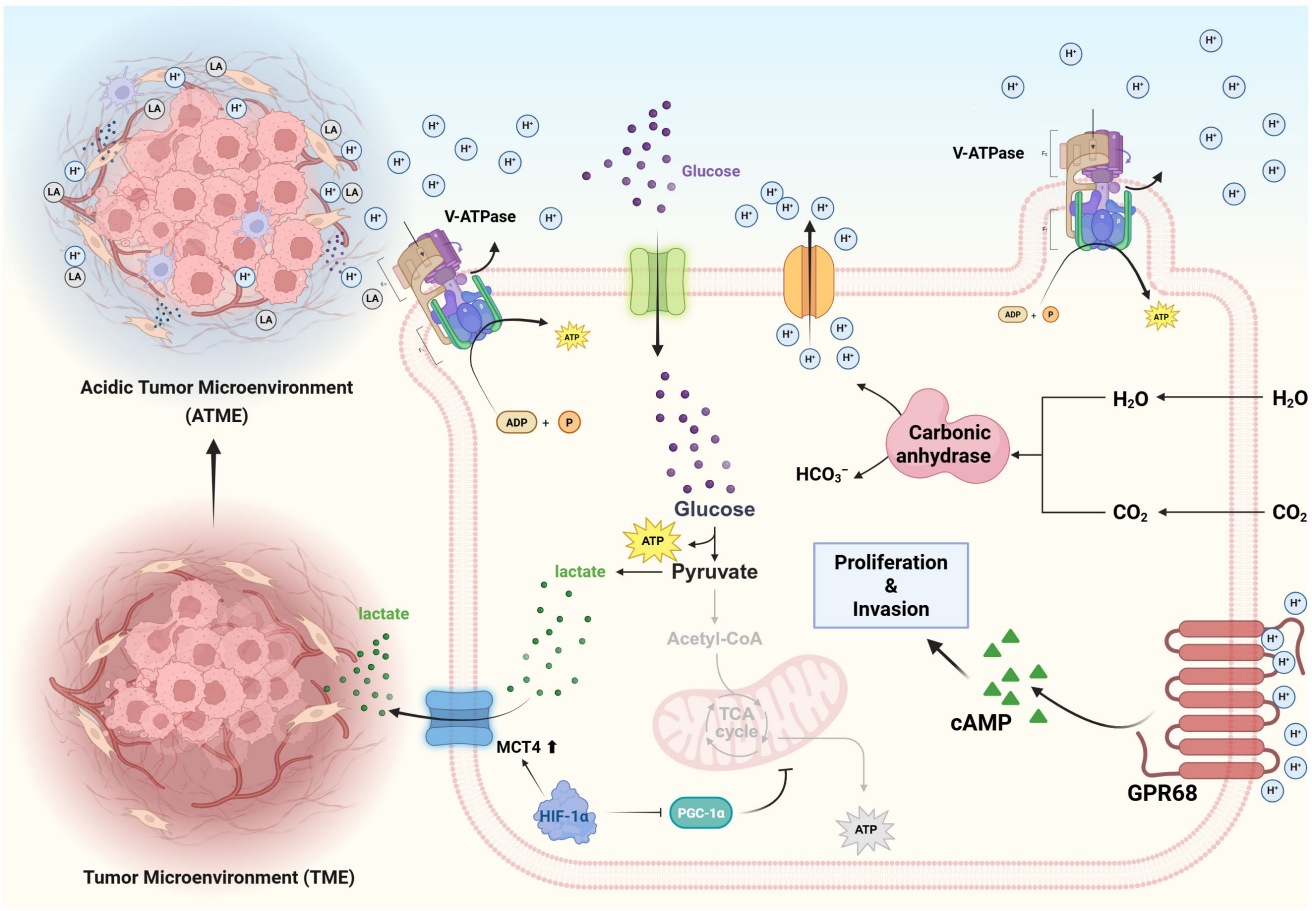
Tumor cells undergo metabolic reprogramming (e.g., enhanced glycolysis) to produce large quantities of acidic metabolic byproducts such as lactic acid. These cells extrude excess H^+^ to the extracellular space via monocarboxylate transporters (MCTs), proton pumps (V-ATPase), and carbonic anhydrase IX (CA IX), resulting in a markedly acidic tumor microenvironment (pH ~6.5–6.9). Additionally, tumor cells express acid-sensing receptors on their surface, primarily including acid-sensing ion channels (ASICs) and proton-sensing G protein–coupled receptors (GPCRs). These receptors are activated under low pH conditions, initiating pro-invasive and pro-proliferative signaling pathways that further enhance tumor cell invasiveness and drug resistance.

In summary, the ATME is far more than just a stressful condition; it is a central orchestrator of tumor malignancy. By coordinately enhancing tumor cell motility, dismantling antitumor immunity, and recruiting stromal cells as allies, the acidic niche establishes a self-reinforcing ecosystem that fosters aggressive disease. This understanding highlights why neutralizing the ATME or disrupting its signaling pathways holds therapeutic promise. The following section will explore how this acidic niche specifically interacts with and sustains the most formidable cellular actors within tumors: cancer stem cells (**Figure 1**).

## 3 The ATME-CSC axis: a symbiotic cycle of state induction and niche engineering

### 3.1 Comparison of metabolic characteristics between normal stem cells and cancer stem cells

To gain a deeper understanding of the metabolic reprogramming of CSCs, it is essential to examine its similarities and differences compared to the metabolic characteristics of normal stem cells (NSCs). NSCs typically rely heavily on the glycolytic pathway to meet their energetic demands (41). This metabolic preference not only facilitates survival within the hypoxic stem cell niche but, more importantly, significantly reduces the generation of reactive oxygen species (ROS)—the byproducts of mitochondrial oxidative phosphorylation (OXPHOS). Consequently, this protects the stem cells from DNA damage and maintains their pluripotency (42).

Similar to normal stem cells, cancer stem cells (CSCs) also exhibit a strong glycolytic phenotype, which enables them to survive in the hostile, hypoxic regions of the tumor microenvironment (TME) and drive tumorigenesis (43). However, the core distinction between CSCs and NSCs lies in their remarkable metabolic plasticity. As described by Stouras et al. (44), CSCs are not strictly confined to glycolysis; instead, they can dynamically switch between glycolysis and OXPHOS in response to fluctuations in oxygen and nutrient availability within the microenvironment (45). Furthermore, CSCs display extensive metabolic dysregulation across other pathways, including lipid (46), amino acid (47), and iron metabolism (48). This high degree of metabolic flexibility serves not only as the biological foundation for maintaining CSC stemness, but also as a critical mechanism enabling them to evade conventional therapeutic interventions, thereby fueling tumor progression and metastasis.

### 3.2 Cancer stem cells: a plastic state governed by the microenvironment

CSCs represent a subpopulation within tumors that are widely regarded as the principal cellular drivers of tumor initiation, therapeutic resistance, relapse, and metastatic spread. Historically, CSCs were conceptualized within a hierarchical model, where a small, static subset of cells, defined by specific surface markers (e.g., CD44, CD133) or functional assays (e.g., Aldefluor activity, sphere formation), possesses the exclusive capacity for self-renewal and tumor propagation (49). This model provided a crucial framework for understanding tumor heterogeneity. However, advances in single-cell technologies have revealed a far more complex and fluid picture. Single-cell RNA sequencing studies demonstrate profound transcriptional heterogeneity within marker-defined CSCs populations, comprising multiple distinct sub-states (50). This heterogeneity is not simply noise but rather signifies a deeper underlying property of CSCs: their phenotypic plasticity. Such plasticity allows CSCs to dynamically adapt to changing microenvironments and therapeutic pressures, underscoring the need for a more nuanced understanding of CSCs behavior.

Emerging evidence now strongly supports the view that “stemness” is not a fixed, cell-autonomous identity, but rather a dynamic and reversible cellular state that can be acquired, maintained, or lost in response to extrinsic signals from the TME (2). Differentiated, non-stem cancer cells can undergo dedifferentiation to re-acquire stem-like properties under specific conditions, such as exposure to therapeutic stress, hypoxia, or—as central to this review—extracellular acidosis (51). This model, centered on plasticity, reframes CSCs not as a rigidly defined cellular entity, but as a context-dependent functional state that can be adopted by a broader pool of tumor cells. Consequently, the CSCs pool is dynamic, constantly shaped and reshaped by microenvironmental cues.

It is within this modern paradigm that the role of the ATME gains critical importance. If the CSCs state is malleable and induced by external signals, then the ATME, with its characteristic low pH and elevated lactate, emerges as a potent regulator. We will explore how acidosis and its associated metabolites act as key microenvironmental signals to induce, maintain, and reinforce the plastic CSCs state, primarily through metabolic reprogramming and epigenetic remodeling, thereby cementing a vicious cycle that drives tumor aggressiveness and treatment failure.

### 3.3 Forging the stem cell state: how acidity drives CSCs generation and maintenance

If the CSCs state is a plastic condition that can be imposed by the microenvironment, then the ATME serves as one of its most potent drivers. Beyond exerting passive selection pressure, the ATME—through the combined effects of low extracellular pH and the accumulation of metabolites like lactate—actively reprograms cell fate. It achieves this by directly modulating the epigenetic landscape and activating core stemness-sustaining signaling pathways, thereby “writing” and locking in the CSC program.

#### 3.3.1 Epigenetic reprogramming: the lactylation nexus

A seminal link between the metabolic state of the ATME and the CSC epigenome is provided by the discovery of histone lysine lactylation (Kla). This finding elevated lactate from a mere metabolic byproduct to a direct precursor for epigenetic modification. Intracellular lactate can be converted to lactyl-CoA, which serves as a substrate for histone lactylation, creating a direct mechanistic bridge from glycolytic flux to chromatin regulation (7). Importantly, lactylation marks, such as H3K18la, are enriched at the promoters of key pluripotency and stemness-associated genes, including OCT4, SOX2, and NANOG, directly driving their transcriptional activation (8). This mechanism transforms the ATME into a sustained stemness signal. For instance, in glioblastoma, histone lactylation upregulates the long non-coding RNA LINC01127, which promotes glioma stem cell self-renewal and tumor progression via the MAP4K4/JNK pathway (52). In hepatocellular carcinoma, lactate-mediated lactylation at histone H3K56 and on the metabolic enzyme ALDOA enhances the stemness of liver CSCs and accelerates tumor progression (53). In colorectal cancer, hypoxia-driven lactate accumulation induces H3K18 lactylation, which activates stemness gene expression and promotes CSC properties (54).

Although histone lactylation serves as a core epigenetic switch, it is crucial to recognize that the ATME-driven “lactylome” extends far beyond chromatin. Localized high concentrations of lactate also drive lactylation of multiple key non-histones, directly reshaping cellular stress responses (55), motility (56), metabolic flux (57), and even microenvironmental immune communication at the post-translational modification level (58). This comprehensively reinforces the malignant state of CSCs (59). This non-histone regulation proves particularly ingenious when cells evade DNA damage induced by chemotherapy. Recent studies reveal that lactylation exerts a highly coordinated, pro-malignant bidirectional regulation on the DNA damage response network: On one hand, lactylation of tumor suppressor p53 diminishes its liquid-liquid phase separation (LLPS) capacity, structurally impairing its DNA-binding and transcriptional activation functions, thereby directly crippling pro-apoptotic damage responses (60). on the other hand, under DNA damage stimulation, the lactylation signal precisely “arms” the core molecular machinery mediating homologous recombination (HR) repair—the MRN complex. CBP not only catalyzes lactylation of MRE11 at Lys673 to dramatically enhance its DNA-binding capacity (61), high lactate concentrations also drive critical lactylation of NBS1 at Lys388 (K388). NBS1 lactylation is essential for the assembly of the intact MRE11-RAD50-NBS1 (MRN) complex and for directing efficient aggregation of HR repair proteins to DNA double-strand break (DSB) sites (62). This synergistic modification of repair complexes enables tumor cells to maintain genomic stability under chemotherapy stress, conferring profound drug resistance (conversely, inhibiting CBP or LDH blocks this process and restores drug sensitivity). Beyond nuclear DNA repair mechanisms, non-histone lactylation extensively remodels cytoplasmic functions and the immune microenvironment, with the cytoskeletal protein MOESIN serving as a key hub. In tumor cells, MOESIN lactylation directly confers enhanced motility and invasiveness (63). Within the microenvironment’s immune cells, lactate modifies the Lys72 (K72) site of MOESIN protein, becoming a key driver of regulatory T cells (Treg) generation (64). Meanwhile, extensive lactylation of key metabolic enzymes—including glycolytic and TCA cycle enzymes—establishes potent positive feedback loops that lock cells into metabolic programs adapted to acidic stress (65). These non-histone lactylation events synergize with epigenetic reprogramming to orchestrate a comprehensive, multi-layered adaptation of tumor cells to the ATME (66). Finally, widespread lactylation of key metabolic enzymes establishes intricate metabolic positive feedback loops. Taking the glycolytic core enzyme M2-type pyruvate kinase (PKM2) as an example: High lactate concentrations significantly enhance PKM2 lactylation. This modification spatially inhibits PKM2 disassembly from tetramers to dimers, thereby robustly sustaining its high pyruvate kinase catalytic activity and markedly reducing its nuclear translocation (67). This lactylation of the metabolic enzyme itself locks cells into metabolic programs adapted to acidic stress. In summary, these non-histone lactylation events synergistically interact with epigenetic reprogramming to comprehensively promote tumor adaptation to the acidic microenvironment and construct a robust immunosuppressive barrier.

The acidic milieu regulates other critical epigenetic layers beyond lactylation. Intracellular pH fluctuations can influence histone acetylation dynamics; a decrease in pHi activates histone deacetylases (HDACs), leading to global deacetylation and proton export, a process implicated in cell survival under acidosis and in maintaining stemness factor expression (68). This dynamic interplay between reduced acetylation and enhanced lactylation reveals a profound underlying biochemical mechanism: competitive acylation kinetics. The epigenetic “writer” p300/CBP exhibits substrate promiscuity, utilizing both acetyl-CoA and lactyl-CoA as modification substrates (69). In normoxic, well-differentiated cells, the mitochondrial tricarboxylic acid (TCA) cycle operates efficiently, generating abundant cytoplasmic acetyl-CoA. This drives p300/CBP to primarily execute classical histone (or non-histone) lysine acetylation. However, in the hypoxic and ATME, impaired mitochondrial function drastically reduces acetyl-CoA production (70). Concurrently, abnormally enhanced glycolysis triggers massive lactate accumulation, which is converted into lactyl-CoA (66). This metabolic reconfiguration triggers a dramatic inversion of the intracellular “lactyl-CoA/acetyl-CoA ratio,” which serves as a fundamental metabolic switch determining cellular fate: When lactyl-CoA concentrations surge, it gains competitive advantage within the catalytic pocket of p300/CBP, forcing the enzyme to undergo substrate switching (71). This results in enhanced lactylation activity while acetylation is competitively inhibited. This imbalance directly triggers profound epigenetic remodeling. At promoter regions of key pluripotency genes like OCT4, lactylation marks (e.g., H3K18la) competitively occupy these critical sites, directly replacing or overwriting existing acetylation marks (72). This elucidates a competitive epigenetic occupancy mechanism, providing a profoundly detailed framework for how “the ratio of metabolites in the local microenvironment directly translates into potent transcriptional activation of tumor stemness programs.” Furthermore, metabolite levels shaped by the ATME, such as α-ketoglutarate (α-KG) and S-adenosylmethionine (SAM), modulate the activity of DNA and histone demethylases (e.g., TET, JmjC-domain proteins) and methyltransferases, respectively. These changes collectively shape an epigenetic landscape permissive for the induction and maintenance of the CSC state (73, 74).

#### 3.3.2 Activation of stemness-sustaining signaling pathways

In parallel with reshaping the epigenome, the ATME directly engages and activates conserved signaling pathways that are fundamental to stem cell biology. Extracellular acidosis can act as a proximal signal that triggers these pathways, often bypassing canonical ligands. A key mechanism is the induction of EMT, a process intimately linked with the acquisition of stem-like traits. Low pH downregulates microRNAs such as miR-193b-3p, leading to the derepression and activation of the TGF-β signaling pathway, a master driver of EMT (30). Acidosis also directly upregulates the expression of core EMT transcription factors like TWIST and ZEB, which repress epithelial genes and enhance motility (29). Beyond TGF-β, the ATME activates other developmental pathways critical for CSC self-renewal. For example, adaptation to acidic conditions in pancreatic cancer enriches populations of cells with active Wnt/β-catenin signaling and high expression of CSC markers like ALDH1, driving metastatic capacity (75). Similarly, acidosis has been shown to downregulate peroxisome proliferator-activated receptor δ (PPARD), which relieves transcriptional repression on the stemness factor SOX2, thereby enhancing CSC properties in colorectal cancer models (76).

#### 3.3.3 Physicochemical cross-linking: synergistic role of acidosis and matrix stiffness in CSC maintenance

Interestingly, the ATME maintains CSCs status not solely through chemical signaling but also profoundly orchestrates the physical properties of the microenvironment. Recent studies reveal that extracellular acidosis serves as a potent trigger for activating matrix metalloproteinases (e.g., MMP1) and other matrix remodeling enzymes (77). This acidic stress induces a dynamic cycle of “collagen degradation” and “CAF-driven collagen deposition” within the TME (78). While MMPs degrade the ECM, partially removing physical barriers to cell migration, this persistent matrix remodeling paradoxically leads to abnormal cross-linking of collagen fibers, ultimately resulting in a significant increase in overall tissue stiffness (79).

This abnormally elevated ECM stiffness serves as a critical mechanical cue. By triggering mechanotransduction networks, this physical alteration directly induces profound crosstalk with the Hippo–YAP/TAZ signaling pathway: the stiffened matrix strongly promotes nuclear translocation and activation of YAP/TAZ, the dominant mechanosensitive transcriptional coactivators (80). Once nuclearized, YAP/TAZ directly transactivates target genes critical for self-renewal and cell survival, comprehensively triggering stemness reprogramming. In uveal melanoma and pancreatic cancer, matrix stiffness mediates Ca²^+^ influx through the mechanosensor PIEZO1, activating the DOT1L or DCLK1-PIP5K1A axis. This induces expression of stemness genes like NANOG and SOX2, enhancing chemotherapy resistance. Inhibiting Ca²^+^ signaling or DOT1L/DCLK1 alleviates stiffness-induced stemness (81). In bladder cancer, matrix stiffness enhances tumor stemness by increasing nuclear pore size and upregulating Lamin A/C, thereby promoting β-catenin nuclear translocation and activating Wnt signaling (82). Thus, acidosis (toxicity in the chemical dimension) and ECM stiffness (rigidity in the physical dimension) function as a tightly coupled “physico-chemical” axis, synergistically and robustly locking tumor cells onto a highly plastic, extremely malignant CSC trajectory.

These activated signaling cascades do not operate in isolation. They engage in extensive cross-talk with the epigenetically reprogrammed landscape. For instance, β-catenin can recruit histone acetyltransferases like p300 to stemness gene loci (83), while TGF-β signaling can influence DNA methylation patterns (84). This creates a synergistic, self-reinforcing regulatory network. The ATME, therefore, acts as a master switch, co-opting both the “hardware” (epigenome) and the “software” (signaling networks) of the cell to establish and stabilize the plastic, therapy-resistant CSC state.

### 3.4 Metabolic plasticity: the engine of CSC adaptation within the acidic niche

The metabolic characteristics of CSCs have been a subject of considerable debate: early studies often depicted them as highly reliant on glycolysis (the Warburg effect), whereas growing recent evidence underscores the critical role of OXPHOS in maintaining their stemness and therapy resistance (85, 86). These seemingly contradictory observations can now be reconciled through a central concept—CSCs do not adhere to a fixed metabolic program but possess intrinsic metabolic plasticity (87). This plasticity enables them to dynamically reprogram their metabolic pathways, such as shifting between glycolysis and OXPHOS, in response to local nutrient availability, oxygen tension, and therapeutic pressures. It thereby constitutes a cornerstone of their adaptability to hostile conditions and their capacity to drive tumor relapse and metastasis (88). Notably, this metabolic flexibility is not autonomous but is profoundly shaped by the tumor microenvironment. Here, the ATME acts as a central regulator (5). Acidosis not only directly enriches slow-cycling, stem-like cell subpopulations through selective pressure (75), but also provides instructive cues for metabolic rewiring in CSCs by influencing nutrient distribution and utilization—for instance, via mechanisms such as cell-programmed nutrient partitioning (89). Thus, by harnessing the metabolic plasticity of CSCs, the ATME creates a decisive condition that allows them to survive and proliferate within a heterogeneous and often hostile microenvironment.

The specific metabolic mode adopted by CSCs is context-dependent, shaped by dynamic microenvironmental cues such as nutrient and oxygen availability, bioenergetic demands, and, crucially, extracellular pH (17). In contexts favoring rapid proliferation and biosynthesis—such as within relatively nutrient-replete or growth factor-rich niche sub-regions—CSCs may upregulate glycolysis. This metabolic state supports the anabolic requirements of cell division by providing carbon skeletons for nucleotide, lipid, and amino acid synthesis (90). This glycolytic phenotype has been robustly linked to stemness in specific contexts. For instance, glioblastoma stem cells (GSCs) residing in the perivascular niche utilize high-rate glycolysis to maintain their stemness and tumorigenic capacity, a process often driven by oncogenic signaling pathways like AKT (91). Similarly, ALDH+ breast cancer stem cells have been shown to exhibit enhanced glycolytic flux, which is functionally required for their clonogenic growth and tumor initiation (6). Conversely, under stress conditions—including acidosis, nutrient deprivation, quiescence, or therapeutic assault—CSCs frequently enhance their dependence on OXPHOS. This mode offers superior ATP efficiency and supports redox homeostasis, which is critical for long-term survival, stress resistance, and immune evasion (92). Importantly, the ATME itself can actively induce a shift toward OXPHOS dependency. In glioma stem-like cells, acidic stress reprograms energy metabolism toward mitochondrial respiration via the CYP24A1/vitamin D signaling pathway (93). Furthermore, lactic acidosis, a hallmark of the ATME, has been shown to force cancer cells to switch from glycolysis to OXPHOS dependence, thereby increasing their sensitivity to OXPHOS inhibitors (94).

The true advantage conferred by metabolic plasticity lies not in the dominance of one pathway over the other, but in the capacity to flexibly switch between them. This “metabolic flexibility” allows CSCs to meet fluctuating bioenergetic and biosynthetic demands while maintaining the low reactive oxygen species (ROS) levels and cellular quiescence often associated with therapy resistance (95, 96). It explains how the same CSC population can appear glycolytic in one experimental setting and OXPHOS-dependent in another. Ultimately, the ability of the ATME to modulate this plasticity—by providing signals (low pH, lactate) that can tip the balance between metabolic states—positions it as a central regulator of CSC fitness. Targeting this adaptive machinery, rather than focusing on a single metabolic pathway, may therefore be a more effective strategy for eradicating this resilient cell population.

### 3.5 The vicious cycle: CSCs as active architects of their acidic niche

The interplay between the ATME and CSCs is not a simple unidirectional process, in which the former merely shapes the latter; rather, it forms a self-reinforcing vicious cycle. CSCs are far from passive occupants of the acidic niche; instead, they act as active architects, deploying specialized molecular machinery to intensify local acidosis and reprogram the surrounding stroma, thereby stabilizing and amplifying the very conditions that sustain their maintenance and competitive dominance.

#### 3.5.1 Active acidification: CSCs as proton export hubs

Emerging evidence underscores that CSCs are not merely passive inhabitants but active architects of the ATME. They deploy a specific molecular arsenal to directly catalyze extracellular acidification, thereby engineering a self-reinforcing niche that favors their own maintenance and excludes competing cell types (19).

To preserve intracellular pH homeostasis while persisting in an acidic milieu, CSCs often upregulate key proton- and lactate-export machineries, including the monocarboxylate transporter 4 (MCT4), the vacuolar H^+^-ATPase (V-ATPase; in some tumors showing functionally relevant enrichment toward the plasma membrane), and the hypoxia-inducible, extracellularly active carbonic anhydrase IX (CAIX). MCT4 mediates the coupled efflux of lactate and H^+^; V-ATPase promotes extracellular acidification by exporting cytosolic H^+^ and has been linked to invasive behavior and therapy resistance; and CAIX catalyzes extracellular CO_2_​ hydration to generate H^+^, thereby further acidifying the local milieu while facilitating bicarbonate handling that supports intracellular buffering (97, 98). Importantly, these pH homeostasis regulators are not merely “housekeeping” components. For example, CAIX has been reported to influence the LIN28/let-7 axis, contributing to tumor-cell adaptation to hypoxic/metabolic stress and coupling pH control to metabolic rewiring and stemness-associated programs (98).

Collectively, these processes can form a feed-forward loop: CSC-enriched regions may become hotspots of acid production and proton/lactate export, sustaining and deepening a hyper-acidic sub-niche. In turn, this self-generated acidosis can function as a biologically active signal that promotes EMT, resistance to apoptosis, clonogenic capacity, and stemness-associated transcriptional programs, thereby further stabilizing a stem-like/plastic cell state (5).

#### 3.5.2 Niche remodeling: CSCs as organizers of the immunosuppressive stroma

Beyond direct proton export, CSCs orchestrate a broader stromal remodeling program by secreting a diverse repertoire of signaling molecules that collectively sustain an acidic and immunosuppressive tumor ecosystem. A central mechanism involves the secretion of chemokines such as CCL2, which potently recruits circulating monocytes to the tumor microenvironment (99). Exposed to instructive cues within the CSC-enriched niche, including elevated lactate levels and other soluble factors, these monocytes preferentially differentiate into tumor-associated macrophages (TAMs) with a profound tissue-repair and pro-tumorigenic profile (100). Rather than mounting effective antitumor immunity, such TAMs promote immune evasion, angiogenesis, and extracellular matrix remodeling (101). In parallel, CSC-derived exosomes and soluble mediators activate cancer-associated fibroblasts (CAFs), initiating a reciprocal stromal interaction (102). Upon activation, CAFs undergo metabolic reprogramming and adopt a pro-tumorigenic phenotype. Notably, lactate exported by CSCs can be imported by CAFs, triggering transcriptional programs that further enhance CAF activation and drive the secretion of additional lactate, immunomodulatory cytokines (e.g., CXCL13) (39). This metabolic symbiosis between CSCs and CAFs amplifies both extracellular acidification and stromal-mediated immunosuppression, reinforcing a self-sustaining malignant niche (103).

**Figure 2:**
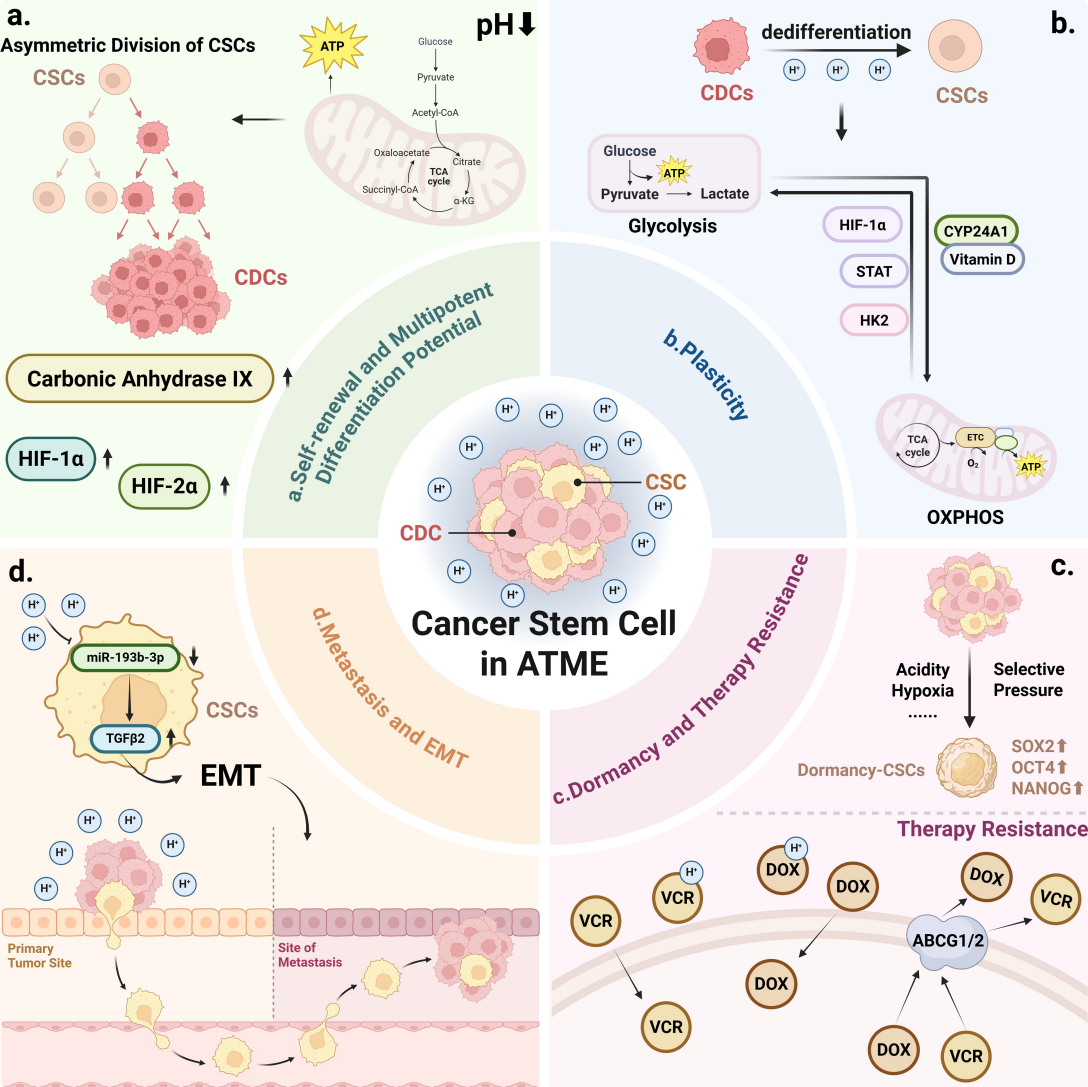
The ATME shapes and reinforces the stemness characteristics of CSCs in multiple ways: **(a)** Low pH conditions help CSCs maintain their self-renewal capacity and multipotent differentiation potential. Through asymmetric division, CSCs can generate differentiated progeny (cancer differentiated cells, CDCs) while remaining undifferentiated under the influence of acid stress factors such as HIF-1α and carbonic anhydrase IX (CA IX), thereby continuously replenishing the tumor cell population. **(b)** Under acidic stress, CSCs can switch their metabolic mode between glycolysis and oxidative phosphorylation (i.e. exhibit metabolic plasticity), for example via the CYP24A1/vitamin D signaling pathway. Furthermore, an acidic environment can induce differentiated tumor cells to regain a stem cell–like phenotype (enhanced cellular plasticity), enabling the tumor to adapt to therapeutic pressure. **(c)** The ATME causes some weakly basic chemotherapeutic drugs to become protonated extracellularly. This charged form of the drug cannot easily penetrate cell membranes, reducing the effective intracellular drug concentration and leading to tumor drug resistance. The ATME also drives a subset of CSCs into a dormant state and upregulates drug-resistance molecules such as ATPbinding cassette (ABC) transporters, rendering these cells resistant to chemotherapy. **(d)** Acidic conditions activate epithelial–mesenchymal transition (EMT)-related pathways (e.g. acid-induced TGF- β signaling), endowing CSCs with greater invasive and migratory capabilities.

In summary, the ATME extends far beyond a passive source of metabolic stress, emerging instead as a definitive and functionally active niche for cancer stem cells CSCs (104). This chapter delineates a dynamic, self-reinforcing partnership in which the ATME—through the combined influence of low extracellular pH and associated metabolites such as lactate—actively programs the CSC state (2). This occurs via the induction of epigenetic reprogramming, most notably through histone lactylation (105), the activation of core stemness-supporting signaling pathways (including Wnt/β-catenin and TGF-β), and the imposition of selective pressures that favor and stabilize metabolic plasticity (106). Conversely, CSCs are not merely shaped by their environment but act as active architects of their niche, reinforcing extracellular acidosis through highly efficient proton-export mechanisms and reshaping the tumor stroma by recruiting and instructing immune and fibroblastic cells to sustain an immunosuppressive, pro-tumorigenic ecosystem (107). Together, this reciprocal feed-forward circuit—ATME ↔ CSC—provides a robust mechanistic foundation for tumor persistence, intratumoral heterogeneity, and therapeutic resistance (108). Consequently, disrupting this vicious cycle represents a compelling and conceptually unified frontier for cancer therapy, a strategy that will be examined in the following sections (**Figure 2**).

## 4 The ATME-CSC axis: executing the malignant program

### 4.1 Co-driver of uncontrolled proliferation: fueling tumor growth

The uncontrolled expansion of tumor mass does not arise from a homogeneous population of proliferating cells. Instead, it is critically driven by a specialized subpopulation forged and maintained by the ATME: CSCs. These ATME-induced CSCs function as the principal regenerative reservoir underpinning both sustained tumor growth and post-therapeutic relapse. Their proliferative contribution is distinctive—not merely in terms of division rate, but in its intrinsic capacity for self-renewal and resistance to therapy, positioning them, together with the ATME, as co-drivers of a persistent and recurrent tumor-propagating pool.

Compelling evidence for this specialized role derives from *in vivo* functional assays, demonstrating that CSCs conditioned by acidosis possess a markedly enhanced tumor-initiating capacity. Across melanoma and pancreatic cancer models, chronic acidic stress selectively expands CSC populations with elevated expression of core stemness regulators (e.g., SOX2, OCT4) and a substantially reduced tumor-initiating cell threshold in immunocompromised hosts, indicative of augmented self-renewal and regenerative potential (75). These findings support an active ATME-driven reprogramming process, rather than passive clonal selection, that enforces a highly aggressive stem-like state.

To sustain this advantage within a hostile tumor milieu, ATME-primed CSCs orchestrate reciprocal niche-signaling programs. In murine mammary tumors, CSCs secrete SHH to activate Hedgehog signaling in CAFs, thereby reinforcing CSC self-renewal and expansion (109). Autocrine and paracrine circuits involving IL-6 and PGE2 further promote CSC survival and proliferation (110). Collectively, these interactions reprogram the surrounding stroma, ensuring a persistent supply of growth factors and extracellular matrix components (e.g., VEGF, HGF) that stabilize a self-reinforcing, pro-proliferative CSC niche resilient to environmental stress (111). Moreover, the proliferative behavior of CSCs is inseparable from their stress-adaptive biology. Metabolic plasticity enables these cells to maintain critical biosynthetic and energetic programs under conditions of nutrient deprivation or hypoxia (87), while heightened resistance to apoptosis and entry into a reversible quiescent (dormant) state permit their survival during cytotoxic therapies that eliminate the majority of rapidly proliferating tumor cells (112). Following therapy withdrawal or microenvironmental remodeling, these therapy-persistent CSCs can re-enter the cell cycle, re-establish tumor growth, and drive disease recurrence (113, 114). Thus, CSC proliferation is tightly integrated with mechanisms of stress tolerance, therapeutic evasion, and long-term persistence (115).

In summary, proliferation governed by the ATME-CSC axis is mechanistically and functionally distinct from that of the tumor bulk. Rather than reflecting indiscriminate hyper-proliferation, it is characterized by persistence, regenerative capacity, and resistance to therapy (116). As a result, conventional anti-proliferative strategies that target rapidly dividing tumor cells frequently spare this critical CSC reservoir, which retains the complete regenerative blueprint required for tumor reconstitution. Accordingly, therapeutic disruption of the ATME-CSC axis represents a necessary strategy to eradicate the cellular root of tumor propagation and to durably prevent disease relapse (117).

### 4.2 Architects of dissemination: orchestrating invasion and metastasis

Metastasis, which accounts for the vast majority of cancer-related deaths, is not a passive or purely stochastic process, but rather an actively orchestrated biological cascade. The ATME and the CSCs it harbors serve as cooperative drivers of this lethal progression (114). Together, the ATME-CSC axis functions as a central regulatory hub, not only facilitating local invasion but also systematically conditioning distant organs for subsequent colonization, thereby executing a highly coordinated, multi-step dissemination program.

The metastatic journey is initiated by local invasion followed by intravasation. Within this context, the ATME directly primes CSCs for enhanced motility and invasiveness. As detailed in Section 2.2.2, acidosis is a potent inducer of EMT, leading to the downregulation of cell-cell adhesion molecules and the upregulation of proteolytic enzymes, such as matrix metalloproteinases (MMPs), which facilitate extracellular matrix degradation (29). CSCs, in turn, potentiate this invasive program by secreting the same EMT-associated factors and further exacerbating local acidification, thereby establishing a self-reinforcing, feed-forward loop that enables tumor cells to breach tissue barriers and gain access to the circulation (30). The most insidious role of the ATME-CSC axis resides in its capacity to remotely orchestrate the formation of the pre-metastatic niche (PMN). Well before the arrival of circulating tumor cells, CSCs emit systemic cues, primarily through tumor-derived exosomes and soluble mediators, that precondition distant organs for metastatic seeding (31). CSC-derived exosomes harbor a defined repertoire of proteins, lipids, and nucleic acids, including microRNAs such as miR-122 and miR-210, which are preferentially internalized by resident cells in the lung, liver, or bone marrow (40). Uptake of these exosomal cargos elicits a coordinated pro-inflammatory and pro-angiogenic response, characterized by increased endothelial permeability, recruitment of bone marrow-derived cells, and suppression of local anti-tumor immunity, thereby establishing a permissive microenvironment that precedes overt metastatic colonization. Importantly, this organotropism is dictated by distinct integrin and glycan signatures displayed on the exosomal surface, which direct exosome homing to specific target organs (118).

Finally, after successfully seeding a distant organ, CSCs must adapt to and ultimately colonize the new tissue, a process that frequently involves entry into a dormant state (119). Their metabolic plasticity and intrinsic stress tolerance are central to this adaptive phase. CSCs can reprogram their energy metabolism to accommodate the initially hostile microenvironment, for example by shifting toward fatty acid oxidation or augmenting glycolytic flux to sustain survival (120). Furthermore, CSCs can adopt a state of therapeutic and proliferative quiescence, thereby evading immune surveillance as well as the cytotoxic effects of adjuvant therapies (121). These dormant disseminated CSCs persist as a latent reservoir for disease relapse, which may be reactivated months or even years later in response to systemic or local niche perturbations, such as chronic inflammation or stromal remodeling (122).

In summary, metastasis should be viewed not as a stochastic complication but as a premeditated, system-level disease process, orchestrated by the ATME-CSC axis. From enabling initial tumor cell escape, to conditioning distant organs for metastatic seeding, and ultimately safeguarding long-term tumor cell survival, this axis coordinates each stage of the metastatic cascade. This conceptual reframing yields an important therapeutic implication: effective anti-metastatic strategies must move beyond the exclusive targeting of circulating tumor cells and instead aim to dismantle both the systemic communication networks (e.g., exosome-mediated signaling) and the adaptive core of metastasis, namely CSC plasticity. Such approaches may be achieved by targeting the ATME itself, CSC-specific vulnerabilities, or their reciprocal interactions, thereby disrupting the hierarchical control architecture that sustains metastatic progression.

### 4.3 Masters of immunosuppression: remodeling the tumor ecosystem

The ultimate success of a tumor depends not only on its intrinsic proliferative capacity but also on its ability to evade and suppress host immune surveillance. The ATME and CSCs act in concert as central regulators of tumor-associated immunosuppression, deploying a coordinated, multi-layered strategy to progressively dismantle effective antitumor immune responses and establish a protective, immunologically “cold” tumor ecosystem. This active reprogramming of the immune landscape constitutes one of the most sophisticated survival strategies employed by tumors, enabling long-term immune escape and sustained disease progression.

#### 4.3.1 Direct sabotage of effector immune cells

The ATME-CSC axis exerts a direct and multifaceted suppressive effect on cytotoxic lymphocyte function. Extracellular acidosis and elevated lactate concentrations profoundly impair the proliferation, cytokine production (e.g., IFN-γ), and cytolytic activity of T cells and natural killer (NK) cells, driving these effector populations into a state of functional paralysis. Mechanistically, lactate induces histone lactylation in CD8^+^ T cells, thereby blunting antigen responsiveness and effector differentiation (123), and directly compromises the metabolic fitness and cytotoxic capacity of NK cells through increased intracellular lactylation (124). Lactate also acts as a signaling metabolite, engaging immune cell receptors such as GPR81 to suppress cellular activation, a pathway conserved across multiple immune cell types (125). Concurrently, CSCs and stromal cells within the acidic niche release a complex array of soluble immunosuppressive mediators. These factors not only directly inhibit effector lymphocyte function but also promote the expansion and recruitment of regulatory T cells (Tregs), whose immunosuppressive activity is further amplified by lactylation-driven upregulation of TNFR2 (126), while shifting macrophage activation toward an SPP1-expressing, highly immunosuppressive phenotype (127). This immunosuppressive circuitry is further reinforced by lactate-reprogrammed, immune-suppressive neutrophils (128), hereby establishing a formidable barrier to effective antitumor immunity.

#### 4.3.2 Disruption of antigen presentation and immune recognition

To evade immune detection, the ATME-CSC axis actively compromises tumor antigen presentation pathways. Acidic stress and CSCs-intrinsic transcriptional and epigenetic programs can downregulate surface expression of major histocompatibility complex class I (MHC-I) molecules on tumor cells, thereby functionally shielding them from CD8^+^ T cell-mediated recognition (129). Furthermore, CSCs deploy a decoy-based immune evasion strategy by secreting extracellular vesicles and exosomes enriched in immune checkpoint ligands, most notably programmed death-ligand 1 (PD-L1). These circulating PD-L1^+^ exosomes engage PD-1 receptors on spatially distant T cells, transmitting potent inhibitory signals that induce systemic T cell dysfunction and exhaustion, even prior to effective tumor infiltration (130).

#### 4.3.3 Metabolic competition and nutrient deprivation

Within the nutrient-deprived TME, CSCs exploit their metabolic plasticity to outcompete immune cells for critical metabolic resources. Their elevated glycolytic flux drives rapid glucose consumption, while increased uptake of glutamine and other amino acids progressively depletes the local nutrient pool. Effector T cells, which depend on these metabolites to sustain activation, proliferation, and effector function, are thereby metabolically starved and functionally compromised—a phenomenon referred to as “metabolic competition” (131). Additionally, CSCs and CSC-associated stromal cells express enzymes such as indoleamine 2,3-dioxygenase (IDO), which catabolizes tryptophan and generates immunosuppressive metabolites, thereby further constraining T cell responses (132).

#### 4.3.4 Recruitment and education of a suppressive stroma

The ATME-CSC axis actively establishes a fortified immunosuppressive barrier by recruiting and functionally reprogramming innate immune cell populations. CSCs secrete chemokines such as CCL2 to drive the mobilization and recruitment of circulating monocytes. Within the acidic, lactate-enriched tumor niche, these monocytes are preferentially instructed to differentiate into TAMs exhibiting a distinctly pro-angiogenic and immunosuppressive phenotype, rather than adopting an anti-tumor, pro-inflammatory activation state (101). Similarly, through the production of chemotactic factors such as CXCL13, the ATME-CSC axis recruits regulatory B cells (Bregs), which further attenuate cytotoxic immune responses and reinforce a tolerogenic tumor microenvironment (39).

**Figure 3:**
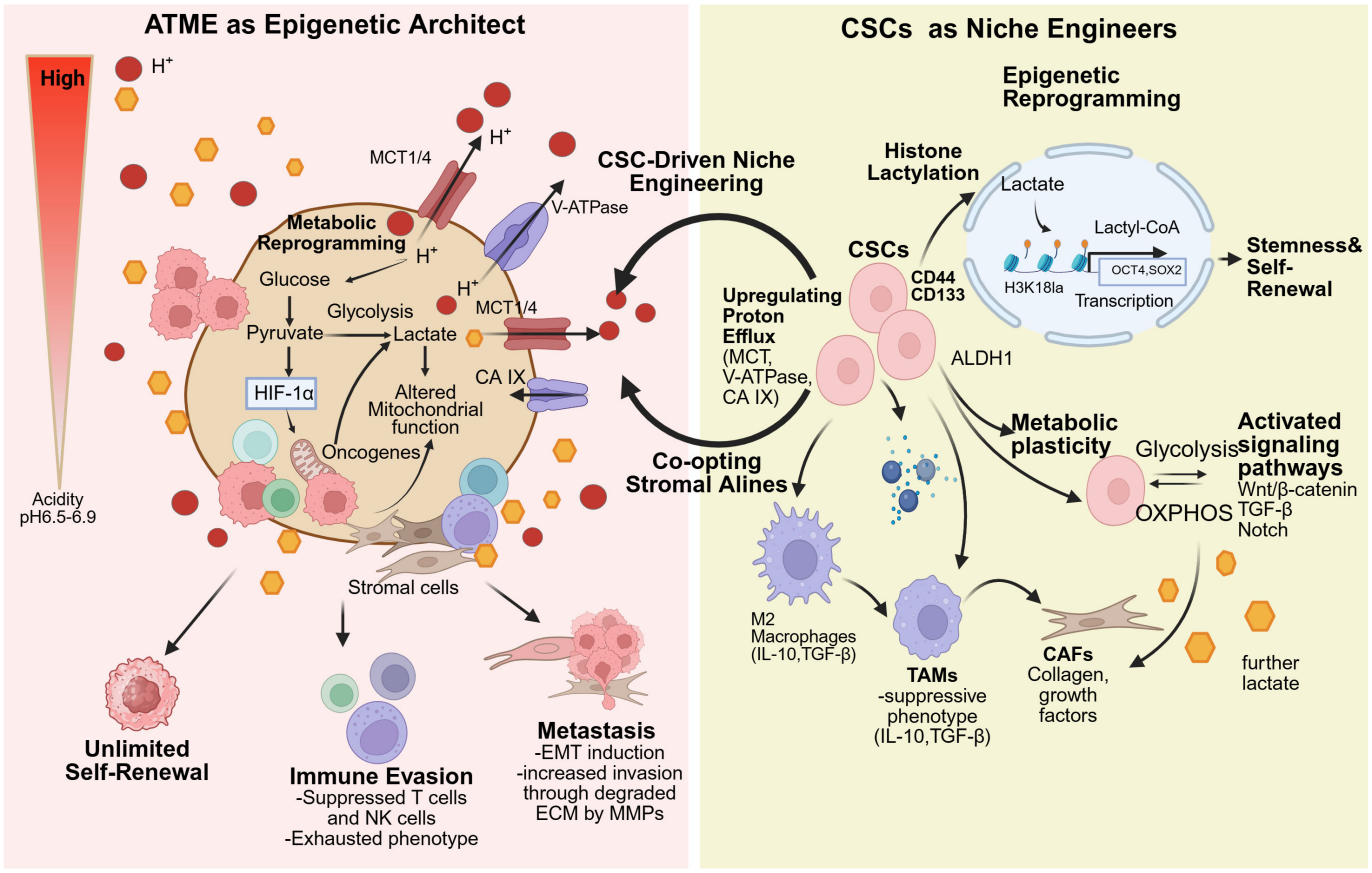
The ATME reinforces CSC stemness through a positive feedback loop integrating metabolic reprogramming, proton efflux, and epigenetic remodeling. The left panel illustrates the ATME as an “epigenetic architect”: enhanced glycolysis generates lactate and H^+^, and proton extrusion via MCT1/4-mediated lactate/H^+^ transport, V-ATPase, and CA IX establishes an extracellular low-pH milieu (≈ pH 6.5–6.9). Acid-stress mediators such as HIF-1α promote maintenance of CSC self-renewal, immune evasion, and metastatic potential. The right panel depicts CSCs as “niche engineers”: CSCs (e.g., CD44^+^, CD133^+^, ALDH1^+^) upregulate MCTs, V-ATPase, and CA IX to further enhance proton efflux and deepen acidification. In parallel, lactate-derived lactyl-CoA drives histone lactylation (e.g., H3K18la) and epigenetic reprogramming, activating transcriptional programs involving OCT4 and SOX2 to sustain stemness and self-renewal. CSCs also exhibit metabolic plasticity (switching between glycolysis and OXPHOS) and activate signaling pathways such as Wnt/β-catenin, TGF-β, and Notch. Lactate and acidity additionally remodel the stromal and immune compartments by driving macrophage reprogramming toward a highly immunosuppressive state and enhancing CAF activity, fostering a tumor-supportive niche that facilitates progression and therapy resistance.

In conclusion, the ATME-CSC axis does not merely evade immune attack; rather, it actively constructs a robust immunosuppressive architecture. By directly suppressing cytotoxic effector cells, concealing tumor cells from immune recognition, outcompeting immune populations for essential metabolic resources, and recruiting and reprogramming a supportive network of immunosuppressive stromal cells, this axis drives the establishment of an immunologically “cold” tumor microenvironment. Such comprehensive immune remodeling represents a fundamental contributor to the limited efficacy of many current immunotherapies. Consequently, overcoming this immune-evasive ecosystem will require rational combinatorial strategies that simultaneously neutralize the acidic niche, target CSC-specific vulnerabilities, and restore durable antitumor immune responses (**Figure 3**).

## 5 Therapeutic disruption of the ATME-CSC axis: from mechanistic insights to clinical translation

### 5.1 disrupting the foundation: neutralizing the acidic niche

As the central driver of the ATME-CSC axis, the acidic tumor microenvironment constitutes a fundamental and therapeutically exploitable target. Therapeutic neutralization of tumor acidity seeks to eliminate the primary selective pressure and signaling stimulus that initiates and perpetuates the malignant cycle. Current therapeutic approaches can be broadly classified into two categories: (i) systemic or local alkalization designed to buffer excess protons, and (ii) targeted inhibition of proton export mechanisms to block acidification at its source.

#### 5.1.1 Systemic and local alkalization: buffering the hostile milieu

This approach seeks to directly increase extracellular pH. Oral sodium bicarbonate (NaHCO_3_) functions as a systemic buffer, neutralizing lactic acid and CO_2_, and has been shown in preclinical models to reduce tumor growth, enhance chemotherapy uptake by alleviating ion trapping, and modestly improve immunotherapy efficacy (133, 134). However, achieving sustained, tumor-targeted alkalinization without causing systemic alkalosis remains a significant clinical challenge. More focused strategies involve proton pump inhibitors (PPIs), such as omeprazole or lansoprazole. By inhibiting the V-ATPase pump on tumor and stromal cell membranes, PPIs can selectively increase intratumoral pH, reverse chemoresistance associated with acidity, and in some models, restore tumor sensitivity to therapy (135, 136). Despite promising preclinical data, the translational efficacy of PPIs as single agents in oncology has been limited, highlighting the complexity of tumor pH regulation and potential compensatory mechanisms (137).

Further complicating the path to clinical application, a growing body of evidence suggests that concomitant use of PPIs can undermine the effectiveness of various anticancer agents. This concern is especially acute for oral drugs whose bioavailability hinges on an acidic gastric environment—such as the tyrosine kinase inhibitors erlotinib, gefitinib, and pazopanib (138). Emerging literature highlights a concerning clinical trend: the concurrent use of proton pump inhibitors (PPIs) with capecitabine yields detrimental impacts on patient survival, underscoring the need for strict therapeutic vigilance. This phenomenon is largely attributed to PPI-mediated gastric achlorhydria; the resultant elevated pH impedes the luminal dissolution and proximal absorption of the prodrug. Such a disruption in absorption ultimately curtails its conversion into the cytotoxic moiety, 5-fluorouracil (5-FU), thereby blunting the intended anti-tumor response (139, 140). Beyond pharmacokinetic interactions, PPIs may also dampen the efficacy of immune checkpoint inhibitors, plausibly through shifts in the gut microbiome that alter antitumor immune responses (141, 142). Given these concerns, recent expert opinion advises against routine PPI co-prescription in cancer patients unless absolutely indicated and suggests considering alternative acid-suppressive strategies (e.g., H2 receptor antagonists or antacids) when required (143, 144).

#### 5.1.2 Precision blockade of proton export: stopping acid at the source

An alternative strategy focuses on inhibiting the molecular machinery that creates the acidic niche, offering a more targeted approach to “source control.” Monocarboxylate transporter (MCT) inhibitors (e.g., AZD3965 targeting MCT1) block the co-export of lactate^−^ and H^+^, a major acidifying pathway. While effective in crippling lactate-dependent tumors in preclinical studies, their clinical development is hampered by on-target toxicities in normal tissues that rely on lactate shuttling (e.g., cardiac muscle) (145). Similarly, targeting carbonic anhydrase IX (CA IX), a hypoxia-inducible enzyme highly expressed on tumor cells, offers high specificity. CA IX inhibitors or antibody-drug conjugates (ADCs) aim to disrupt its dual role in extracellular acidification and intracellular pH buffering (23, 146). The challenge lies in tumor heterogeneity of CA IX expression and its functional redundancy with other CA isoforms.

#### 5.1.3 Challenges and the imperative for combination therapy

It is crucial to recognize that neutralizing the acidic niche, while necessary, is likely insufficient as a standalone therapy. The ATME is not merely a condition but an active instructor that has already epigenetically “imprinted” or reprogrammed the CSC state, fostering traits like therapy resistance and dormancy (147). Simply raising the pH may not erase this deeply wired stemness identity, potentially allowing CSCs to persist or adapt through alternative pathways. Moreover, compensatory upregulation of other pH-regulatory proteins can lead to treatment resistance. Therefore, strategies aimed at disrupting the foundation (alkalinization) must be rationally combined with those designed to eliminate the established CSC population by cutting their metabolic fuel lines or directly erasing their malignant epigenetic program. This multi-pronged approach is essential for collapsing the self-reinforcing ATME-CSC axis.

### 5.2 Cutting the fuel lines: dual targeting of CSC metabolic plasticity

The remarkable metabolic plasticity of CSCs — their ability to flexibly utilize glycolysis, OXPHOS, or both — poses a formidable therapeutic challenge. As detailed in Section 2.3, this plasticity is a core adaptive mechanism, allowing CSCs to survive in the acidic niche and resist therapies that target a single metabolic pathway. Consequently, any effective metabolic intervention must confront this adaptability head-on. Strategies that simultaneously or sequentially inhibit both major energy pathways, or target the master regulators of metabolic switching, are therefore essential to disrupt the fuel supply of the ATME-CSC axis.

#### 5.2.1 Dual-pathway inhibition: a logical but challenging approach

Given the risk of therapeutic escape through metabolic remodeling, the rational combination of glycolytic and OXPHOS inhibitors has gained traction in preclinical research. For instance, combining the glycolytic inhibitor 2-deoxy-D-glucose (2-DG) with the OXPHOS inhibitor lovastatin induces synergistic apoptosis in KRAS-mutant colorectal cancer cells by causing severe energy crisis and AMPK/mTOR pathway inhibition (148). More comprehensive approaches are emerging, such as the use of nanocarriers delivering dual inhibitors. One study employed a hyaluronic acid nanoparticle to co-deliver the NOX inhibitor GKT831 (indirectly disrupting OXPHOS-associated redox balance) alongside radiotherapy (which enhances glycolytic dependency), achieving an 84.7% tumor inhibition rate in a colorectal cancer model by synergistically depleting ATP and increasing DNA damage (149). However, the clinical translation of such aggressive metabolic blockade is fraught with challenges, primarily due to the high energy demands of vital normal tissues like the heart and brain, which could lead to dose-limiting toxicities.

#### 5.2.2 Targeting the command center: disabling metabolic plasticity itself

A more sophisticated strategy aims not at the metabolic pathways themselves, but at the upstream regulators that orchestrate the plasticity. By disabling the cell’s ability to switch its metabolic mode, this approach could sensitize CSCs to metabolic stress or existing inhibitors. Promising targets include (1): Hypoxia-Inducible Factors (HIFs): As master regulators of the glycolytic switch, HIF-1α/2α or metabolic enzyme inhibitors (e.g., DFF332, currently in clinical trials) could prevent the induction of glycolysis in response to the acidic/hypoxic niche, potentially locking cells in a vulnerable metabolic state (150–152). (2) Mitochondrial Dynamics Regulators: Proteins like OPA1, which control mitochondrial fusion and cristae structure, are critical for efficient OXPHOS. Inhibiting OPA1 (e.g., with MYLS22) disrupts mitochondrial function and could prevent the adaptive shift to OXPHOS under stress (153). (3) Oncogenic Transcriptional Hubs: Factors like MYC and β-catenin concurrently drive the expression of glycolytic and mitochondrial biogenesis genes. Targeted degradation of these proteins (e.g., with Physiosporin) could dismantle the coordinated metabolic program (154).

Targeting these “plasticity switches” represents a frontier in cancer metabolism therapy, potentially offering a broader therapeutic window than direct ATP pathway inhibition, but their efficacy and specificity in eliminating CSCs within the TME require extensive validation.

#### 5.2.3 Critical analysis and delivery strategies

The primary hurdle for all metabolic therapies is the therapeutic index. The fundamental necessity of glycolysis and OXPHOS for normal cellular homeostasis means that systemic inhibition carries significant risk. To overcome this, advanced tumor-targeted delivery systems are indispensable. Nanoparticles, liposomes, or antibody-drug conjugates (ADCs) designed to selectively accumulate in tumors—via the enhanced permeability and retention (EPR) effect, whereby macromolecules preferentially extravasate through leaky tumor vasculature and are retained due to impaired lymphatic drainage—can concentrate these potent metabolic disruptors at the disease site, sparing critical organs (155–157). Furthermore, leveraging the unique features of the ATME itself—such as developing pH-sensitive nanocarriers that release their cargo specifically in acidic regions—could provide a second layer of selectivity, precisely reprogramming the metabolic and immune microenvironment within the tumor tissue (158).

To bridge the gap between preclinical findings and clinical application, and to provide a current landscape of therapeutic strategies targeting the acidic tumor microenvironment (ATME) and cancer stem cell (CSC) markers, we have summarized a selection of actively recruiting or ongoing clinical trials from 2024 to 2026 (**Table 1**). This compilation highlights the diverse modalities under clinical investigation, underscoring the translational momentum and therapeutic potential of these targets in oncology.

**Table 1:** Representative active clinical trials targeting ATME or CSC markers (2024–2026).

Target	Intervention	Mechanism of action	Indication	Clinical phase	ClinicalTrials.gov ID (Status)
MCT1 (Monocarboxylate Transporter 1)	AZD3965	AZD3965 is an oral, selective MCT1 inhibitor that blocks tumor cells from utilizing lactate as metabolic fuel, thereby inhibiting tumor growth in acidic microenvironments.	Advanced Solid Tumors, Diffuse Large B Cell Lymphoma, Burkitt Lymphoma	Phase I	NCT01791595 (Completed/Active Follow-up)
Hedgehog (SMO)	Sonidegib + Blue-Light Photodynamic Therapy	Sonidegib is a Smoothened (SMO) antagonist that acts on the SMO receptor in the Hedgehog pathway, binding to the drug-binding pocket of SMO to block downstream signaling.	Multiple Basal Cell Carcinomas	Phase I	NCT06623201 (recruiting)
Hedgehog (SMO)	Sonidegib + Cemiplimab(Anti-PD1- antibody)	Sonidegib inhibits Hedgehog signaling by blocking SMO, while Cemiplimab is a PD-1 antibody.	Advanced Basal Cell Carcinoma	Phase II	NCT04679480 (Active, not recruiting)
Hedgehog (SMO)	Personalized CSC High-Throughput Screening + Standard Chemoradiotherapy	Sonidegib’s Hedgehog inhibition has been applied in basal cell carcinoma; regarding trials targeting CSCs, NCT05380349 is a personalized therapy trial utilizing patient-derived cancer stem cell lines for high-throughput drug screening. It selects up to three FDA-approved drugs for each newly diagnosed glioblastoma patient, administered in combination with standard radiotherapy and temozolomide chemotherapy. This trial targets the unique vulnerabilities of tumor stem cells without fixing on a single drug; therefore, Sonidegib is not administered to all participants.	Newly Diagnosed Glioblastoma	Early Phase I	NCT05380349 (recruiting)
CTLA-4 (Acidic pH-Sensitive Antibody)	Gotistobart (ONC-392)	Gotistobart is an acid pH-sensitive anti-CTLA-4 monoclonal antibody developed by OncoC4. The press release states that this antibody “selectively dissociates at acidic pH, avoiding degradation in lysosomes, thereby enhancing the therapeutic index and enabling selective clearance of regulatory T cells within the tumor microenvironment.” As an immune checkpoint inhibitor, ONC392 enhances antitumor immunity and improves safety.	Advanced Solid Tumors and NSCLC	Phase I/II	NCT04140526(Active, not recruiting)
DLL3 (CSCs marker)	FZ-AD005	FZAD005 is a novel DLL3-targeting antibody-drug conjugate (ADC) composed of the anti-DLL3 antibody FZAA038 linked to the topoisomerase I inhibitor DXd via a dipeptide linker, with reduced off-target toxicity through Fc silencing technology. Preclinical studies indicate DLL3-specific binding, efficient endocytosis, and bypass killing, demonstrating significant antitumor activity *in vivo*.	Advanced Solid Tumor, Small Cell Lung Cancer or Large Cell Neuroendocrine Carcinoma	Phase I	NCT06424665 (recruiting)
DLL3 (CSCs marker)	IBI3009	IBI3009 is a DLL3-targeted antibody-drug conjugate (ADC) that utilizes a topoisomerase I inhibitor platform, targeting DLL3, which is lowly expressed in normal tissues but highly expressed in small cell lung cancer (SCLC) and other neuroendocrine tumors. This ADC is designed to selectively deliver a cytotoxic payload into DLL3-positive tumor cells.	Small Cell Lung Cancer	Phase I	NCT06613009 (recruiting)
DLL3 (CSCs marker)	Tarlatamab (AMG 757)	Tarlatamab is a DLL3-targeting bispecific T cell engager (BiTE) that simultaneously binds DLL3 on tumor cell surfaces and CD3 on T cells, thereby recruiting and activating T cells to kill DLL3-positive cancer cells.	Metastatic/Locally Advanced Small-Cell Lung Cancer and Other Poorly Differentiated Neuroendocrine Carcinomas	Phase II	NCT07016230 (recruiting)

### 5.3 Erasing the malignant program: epigenetic reprogramming as a core strategy

While neutralizing the niche and disrupting metabolism target the enabling conditions of the ATME-CSC axis, a more fundamental strategy aims to directly erase the malignant “operating system” itself—the epigenetic program that defines the CSC state. As established in Section 2.2.1, metabolites from the ATME, particularly lactate, write a pro-stemness signature onto the chromatin via modifications like histone lactylation. Reversing this acquired epigenetic identity represents a potentially curative “reprogramming” approach to eliminate CSCs.

#### 5.3.1 Targeting the lactylation axis: from writers to erasers

The lactylation pathway offers two key nodal points for intervention: preventing the mark from being written, or actively removing it.

Inhibiting Lactylation “Writers”: This upstream strategy seeks to deplete the substrate or inhibit the enzymes that catalyze lactylation. Lactate dehydrogenase A (LDHA) inhibitors (e.g., GSK2837808A) reduce the production of lactate, the essential precursor for lactyl-CoA (159). In parallel, inhibitors of p300/CBP, the primary histone acetyltransferases also responsible for catalyzing histone lactylation, can directly block the deposition of lactyl marks on chromatin (7, 160).

Preclinical studies show that such inhibition attenuates the expression of stemness genes and impairs CSC self-renewal. For instance, lactylation enhances the expression of GCLC in colorectal cancer stem cells via p300 at the H4K12 site, thereby promoting their chemoresistance. Inhibition of p300 or LDHA reduces H4K12 lactylation levels and increases the chemosensitivity of colorectal cancer stem cells (161).

Developing Lactylation “Erasers”: A groundbreaking therapeutic avenue involves identifying and modulating enzymes that remove lactyl marks. While the field is young, certain sirtuin family deacylases (SIRTs) have shown delactylase activity in specific contexts. Furthermore, natural products like Demethylzeylasteral (DML) and Royal Jelly Acid (RJA) have been identified as potent lactylation erasers. DML downregulates oncogenic H3K9la and H3K56la to suppress liver CSC tumorigenicity (162), while RJA inhibits glycolysis and erases H3K9la/H3K14la to block HCC progression (163). These molecules provide proof-of-concept that pharmacological “epigenetic erasure” is feasible.

#### 5.3.2 Integrating complementary epigenetic modalities

Lactylation does not operate in isolation. A comprehensive epigenetic therapy should consider the crosstalk with other modifications. DNA methyltransferase (DNMT) inhibitors (e.g., azacitidine) and TET-family activators can reverse the hypermethylation-mediated silencing of tumor suppressor genes often found in CSCs (164, 165). Conversely, histone deacetylase (HDAC) inhibitors can counteract the global deacetylation promoted by acidosis (166). Furthermore, blocking acetylation “readers” disrupts their downstream functional transduction, offering a highly promising therapeutic window. Nguyen et al. Discovered that lactate’s transcriptional regulation of the core oncogene MYC directly depends on bromodomain-containing protein 4 (BRD4) (2). BRD4 recognizes acetylated histones and recruits transcriptional activation complexes, thereby sustaining high MYC expression levels. In the dynamic transition between cancer stem cells (CSCs) and differentiated cells (CDCs), the BRD4-MYC axis promotes CSCs maintenance and CDCs dedifferentiation, leading to enhanced tumor stemness and increased drug resistance. Therefore, targeting this interaction emerges as a potent strategy to disrupt the “metabolic-epigenetic” link. BRD4 inhibitors (i.e., BET inhibitors such as OTX015 or Pelabresib) competitively displace BRD4 from acetylated histones (167). This intervention effectively disrupts the coupling between ATME-induced lactate signaling and oncogenic transcriptional output, thereby fundamentally depriving CSCs of their MYC-dependent adaptability. This represents a highly promising targeted strategy for preventing tumor recurrence. Rational combinations targeting lactylation alongside these other pathways could achieve synergistic reprogramming, forcing CSCs to exit their stem-like state and lose their tumorigenic potential. Studies reveal a positive feedback loop between the glycolysis-lactylation axis and the transcription factor LHX2, wherein LHX2 further reinforces the neuroendocrine phenotype by upregulating DNMT1 expression. This demonstrates the intertwined relationship between lactylation and DNA methylation mechanisms in driving tumor progression, providing a rationale for the combined targeting of these two epigenetic regulatory axes (168).

#### 5.3.3 The future: precision epigenetic editing

Looking ahead, the ultimate form of epigenetic reprogramming may lie in precision epigenome editing. The foundational concept of fusing CRISPR-dCas9 to epigenetic effectors has rapidly advanced towards therapeutic application. Recent breakthroughs have demonstrated the feasibility of *in vivo* targeted epigenetic modulation, such as locus-specific DNA methylation in human cells, showcasing its direct therapeutic potential (169). Furthermore, these efforts are guided by sophisticated, genome-wide frameworks for mapping gene regulatory networks, ensuring that interventions are precisely targeted to the most critical nodes (170, 171). As the field evolves, it is moving decisively towards achieving quantitative and multiplex control of chromatin states for therapeutic purposes (172). Building on this progress, we can envision designing editors that precisely erase lactylation marks to inactivate the OCT4 or SOX2 promoters, or rewrite methylation patterns to activate differentiation programs. Although significant challenges remain in delivery, specificity, and safety for clinical oncology, this rapidly advancing frontier represents the logical endpoint of a curative strategy aimed at eliminating the malignant epigenetic program at the very root of the ATME-CSC axis.

### 5.4 Integrated strategies and future perspectives: breaking the cycle

The preceding sections have deconstructed the ATME-CSC axis into targetable components. However, the very nature of this self-reinforcing cycle suggests that attacking any single node in isolation is unlikely to yield durable cures. Adaptive feedback and compensatory plasticity will fuel resistance. Therefore, the logical and necessary evolution in therapeutic design is toward rational, multi-layered combinations that cripple adaptive machinery, dismantle the niche, and reprogram cellular identity.

Based on the mechanistic model established throughout this review, the most promising integrative approach can be conceptualized as a “three-pillar” strategy: Niche Neutralization, Metabolic Dual-Inhibition, and Epigenetic Reprogramming. This multi-layered strategy aligns with the evolving therapeutic paradigm of concurrently targeting multiple, interdependent components of the tumor ecosystem to overcome adaptation and resistance (173). The synergy of this framework is paramount:

Pillar 1: Niche neutralization. This pillar posits that weakening foundational tumor-promoting signals can render the microenvironment less hospitable for cancer cells, potentially sensitizing them to therapeutic agents and contributing to the reversal of key pathological stromal characteristics. Compelling evidence for this concept comes from studies targeting lactate-driven signaling and histone lactylation. For instance, in hepatocellular carcinoma, inhibiting histone lactylation via Dihydroartemisinin-mediated YAP1 suppression reprograms the immune landscape, transforming immunologically “cold” tumors into “hot” ones by enhancing CD8+ T cell infiltration and synergizing with anti-PD-1 therapy (174). Similarly, in cervical cancer, tumor cell-derived lactate, amplified by the ICAT/c-Myc-ENO1 axis, induces H3K18 lactylation in tumor-associated macrophages, driving their functional reprogramming toward a highly immunosuppressive phenotype that fosters an immune-evasive niche (175). Furthermore, in pancreatic cancer, lactate secreted by perineural invasion (PNI)-associated cancer-associated fibroblasts is taken up by tumor cells to drive histone H3K18 lactylation, which transcriptionally activates pro-invasive genes (e.g., L1CAM, SLIT1) and directly fuels the PNI process (176). Collectively, these studies demonstrate that lactylation is a central mechanism for reshaping an immunosuppressive, pro-angiogenic, and pro-invasive niche. Targeting this process systemically counteracts these hallmarks of the tumor microenvironment—by reversing immunosuppression, disrupting protumorigenic metabolic symbiosis, and inhibiting invasive programs—thereby neutralizing the supportive niche critical for tumor progression and therapy resistance.

Pillar 2: Metabolic dual-Inhibition. This strategy directly attacks the energetic and biosynthetic flexibility that is a hallmark of cancer cell resilience. The core premise is to achieve superior therapeutic efficacy by simultaneously crippling the production of oncometabolites, such as lactate, and blocking their downstream functional consequences, thereby preventing metabolic escape and driving cells into a vulnerable state. Support for this multi-node intervention is robustly illustrated by studies employing combination therapies that disrupt the lactate axis. In melanoma, the concurrent use of an LDH inhibitor (suppressing lactate generation) with an anti-angiogenic agent (countering lactate-driven vascular promotion) synergistically inhibited tumor growth, exemplifying a direct co-inhibition of the metabolic source and a key pathological output (177). Similarly, in pancreatic cancer, co-targeting the upstream regulator PSMD14 (which stabilizes LDHA) alongside the broader glycolytic/lactylation pathway potently suppressed tumor progression, demonstrating effective multi-pronged inhibition within the same metabolic cascade (178). Beyond classic drug combinations, targeting a critical nexus point can also enact a “dual-inhibition” effect by concurrently reprogramming metabolism and resensitizing cells to treatment. For instance, in pancreatic ductal adenocarcinoma, inhibition of CD147 not only triggered lactylation-dependent ferroptosis but also profoundly sensitized tumors to gemcitabine, achieving both metabolic disruption and chemo-sensitization through a single target (179). Collectively, these examples validate that concerted intervention at both the origin and the functional endpoints of oncogenic metabolic networks—such as the lactate-driven signaling axis—amplifies therapeutic impact by addressing the metabolic vulnerabilities and adaptive plasticity of cancer cells.

Pillar 3: Epigenetic reprogramming. This strategy addresses the root cause of cancer malignancy by targeting to erase or rewrite the oncogenic epigenetic “software” installed by the aberrant tumor microenvironment. It aims to remove stemness-associated epigenetic marks, such as lactylation, to force cancer cells out of their resilient state, potentially leading to differentiation or cell death and providing a definitive therapeutic solution. The metabolite-induced modification of histone lactylation has emerged as a pivotal mechanism in this process, directly translating glycolytic flux into stable pro-tumorigenic gene expression programs. Foundational evidence comes from studies that have defined its core enzymatic machinery, revealing that ACSS2 functions as a lactyl-CoA synthetase and partners with KAT2A as a dedicated lactyltransferase to catalyze histone lactylation, thereby activating oncogenic pathways like Wnt/β-catenin and PD-L1 expression (180). Beyond the mechanism, the discovery of novel histone lactylation sites, such as H4K79la and H4K91la in breast cancer, has uncovered self-reinforcing positive feedback loops. These modifications directly drive the transcription of glycolytic genes (e.g., LDHA, PGK1), thereby locking cells into a perpetual state of metabolic rewiring and epigenetic activation that sustains the malignant phenotype (181). Furthermore, the functional impact of specific lactylation marks is exemplified in colorectal cancer, where mutant KRAS elevates H3K9la levels to enhance chromatin accessibility and transcriptionally upregulate the cholesterol transporter GRAMD1A. This directly links a key driver mutation to a lactylation-mediated epigenetic switch that fuels tumor progression (182). Collectively, these studies demonstrate that lactylation serves as a direct epigenetic conduit, enabling tumor cells to encode sustained metabolic and oncogenic signals into their chromatin landscape. Targeting this reprogramming axis holds the promise of durably resetting the gene expression profile of cancer cells, erasing the “metabolic memory” that underpins their aggressiveness and therapeutic resistance.

This sequential and synergistic logic—disrupt the environment, cut the fuel supply, then erase the malignant program—represents a blueprint for collapsing the ATME-CSC axis comprehensively.

#### 5.4.1 Confronting challenges and developing predictive biomarkers

Translating this ambitious therapeutic framework into clinical practice faces significant, interrelated hurdles. First, profound tumor heterogeneity ensures that not all cells are equally dependent on the lactate-lactylation axis, allowing for Darwinian selection of resistant clones upon treatment pressure (183), Second, therapeutic tolerance will inevitably evolve, potentially through the emergence of new genomic alterations or non-genetic, adaptive persister states (184). Third, effective drug delivery to all relevant cellular compartments—including hypoxic/acidic niches, CSCs, and supportive stromal cells—remains a major pharmacological challenge. Finally, managing the potential long-term on-target and off-target toxicity of combining multiple potent agents targeting core metabolism and epigenetics requires careful consideration. To navigate these challenges and enable precision intervention, the field must concomitantly develop dynamic, axis-centric biomarkers. A promising direction involves liquid biopsy assays capable of detecting lactylation-modified histones in circulating exosomes or nucleosomes (185). Technologically, the feasibility of profiling specific histone modifications from blood has been established by chromatin immunoprecipitation of cell-free nucleosomes (cfChIP-seq) (186). adapting such approaches to monitor lactylation dynamics could provide a minimally invasive surrogate for real-time tumor burden, CSC activity, and pharmacodynamic target engagement.

In parallel, advanced functional imaging techniques are emerging as critical tools. For instance, a direct consequence of glycolytic flux, as established in preclinical methodologies (187). Similarly, hyperpolarized ¹³C-pyruvate MRI, which has demonstrated clinical feasibility for real-time metabolic imaging (188), can provide a dynamic readout by visualizing the conversion of pyruvate to lactate, directly reporting on *in vivo* LDHA activity. Recent advances in deep learning-based dose prediction, such as the work by Huang et al., have demonstrated the feasibility of using AI to model tumor-specific physical parameters. Integrating such approaches with pH-sensitive imaging could enable non-invasive mapping of acidic niches and guide personalized therapeutic interventions (189). The future clinical translation of such multiplexed imaging biomarkers will be critical for intelligent patient stratification, monitoring early molecular response, and detecting emergent resistance mechanisms, thereby guiding adaptive therapy within this novel framework.

#### 5.4.2 Future outlook: from static targets to dynamic ecosystem intervention

The ATME-CSC axis paradigm necessitates a paradigm shift in oncology—from targeting static molecular lesions to therapeutically intervening in a dynamic, adaptive ecosystem. The future lies in developing “smart” therapeutic platforms that respond to the unique features of this malignant niche, such as pH- or enzyme-activated nanoparticles capable of sequential drug release. For instance, A pH-responsive polycarbonate-based nano-platform (PEDH NPs) was developed for sequential drug release in the treatment of non-small cell lung cancer. By combining erlotinib with doxorubicin, it significantly enhanced the anti-tumor efficacy of the drugs (190). Similarly, bispecific molecules that simultaneously engage a CSC marker (e.g., DLL3) and an immune cell (e.g., T-cell via CD3) to redirect cytotoxic activity (191).

Emerging radiotherapy modalities such as FLASH, as investigated by Yang et al., offer the potential to spare normal tissues while maintaining antitumor efficacy. This approach may preserve immune effector cells within the acidic tumor microenvironment, thereby synergizing with immunomodulatory strategies aimed at disrupting the ATME-CSC axis (192).

**Figure 4:**
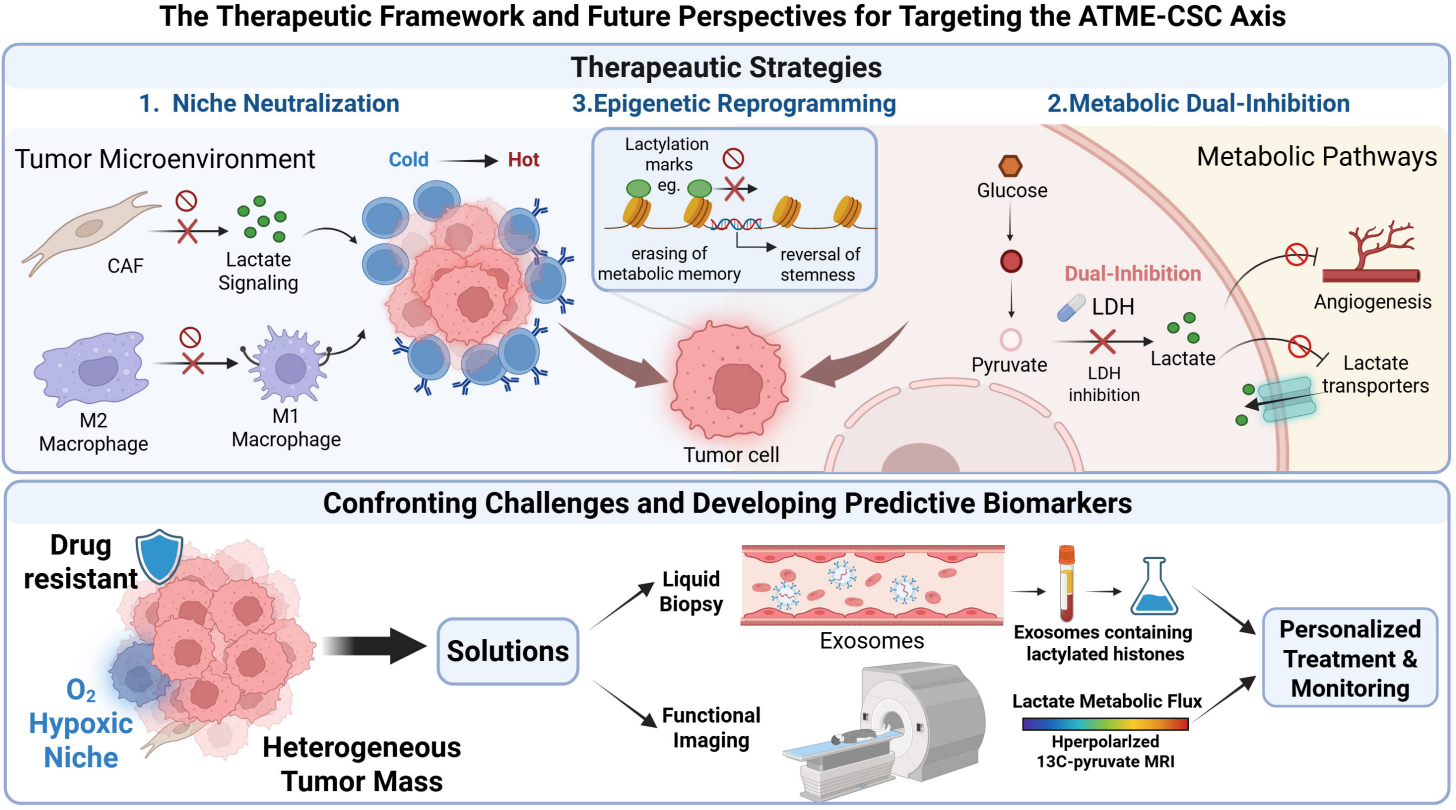
The upper panel summarizes three major intervention avenues (1): Niche neutralization—blocking CAF-derived lactate signaling and remodeling the immune microenvironment to reprogram macrophages from an immunosuppressive state toward a pro-inflammatory, anti-tumor phenotype, thereby converting an immunologically “cold” tumor into a “hot” tumor; (2) Metabolic dual-inhibition—simultaneous suppression of lactate production and transport (e.g., LDH inhibition and blockade of lactate transporters) to reduce lactate accumulation and downstream protumor processes such as angiogenesis; and (3) Epigenetic reprogramming—targeting lactylation and related epigenetic marks to erase “metabolic memory” and reverse stemness-associated phenotypes. The lower panel highlights key translational needs: given therapy challenges posed by heterogeneous niches (e.g., hypoxia), predictive biomarker development integrating blood-based assays/liquid biopsy, metabolic profiling, and imaging readouts can enable personalized treatment selection and longitudinal monitoring.

Furthermore, preclinical testing must evolve beyond conventional cell lines and xenografts. Patient-derived tumor organoids (PDTOs) cultured with autologous immune cells and under controlled physico-chemical conditions (e.g., adjustable pH, oxygen) will be indispensable for modeling the complex ATME-CSC-immune interplay and for personalized evaluation of multi-modal therapeutic combinations (193, 194). By embracing this integrated, ecologically informed perspective, we can move closer to the ultimate goal of not just shrinking tumors but permanently breaking the vicious cycles that sustain them (**Figure 4**).

## 6 Perspectives and future directions

The research synthesized in this review firmly establishes the ATME not as a passive backdrop, but as an active instructive niche and a core component of the ATME-CSC axis—a self-reinforcing engine driving tumor initiation, plasticity, therapy resistance, and metastasis. This paradigm shift, from viewing acidosis as a mere side effect to recognizing it as a central organizer of malignancy, opens transformative avenues for both fundamental research and clinical translation. Moving forward, the field must prioritize research that captures the dynamic reciprocity of this axis and translates mechanistic insights into disruptive therapeutic strategies.

Central to this evolution is the imperative to decode the spatial and temporal dynamics of the axis. Although traditional bulk sequencing has established a molecular link between lactate and histone lactylation, it disrupts tissue architecture and fails to capture the spatial heterogeneity of the TME. Moving beyond static correlations, future studies should aim to map the spatiotemporal architecture of the ATME-CSC axis within intact tumors through the integration of spatial multi-omics technologies (195). By leveraging spatially resolved transcriptomics, metabolomics (e.g., MALDI-MSI, DESI), and epigenomics, researchers can begin to address how lactylation-modified CSC subclusters (e.g., H3K18la) are spatially organized relative to lactate gradients and hypoxic regions. Specifically, the integration of spatial metabolomics (such as MALDI-MSI or DESI mass spectrometry imaging) with high-resolution spatial transcriptomics (such as Stereo-seq, Xenium, or Visium) will be crucial. Theoretically, this integrated map will reveal a unique “Lactylation Zone” situated at the interface between necrosis and hypoxia. Within this niche, MALDI-MSI will directly detect peak lactate concentrations and clusters of acidic pH signals, thereby mapping precise metabolic gradients. Crucially, by overlaying multi-omics data, researchers can validate whether this metabolic gradient highly colocalizes with the spatial enrichment of H3K18la modifications. This spatial resolution will enable testing of a core hypothesis: whether cells with high H3K18la expression within the high-lactate core zone specifically co-express pluripotency factors (e.g., OCT4, SOX2) and drug efflux pumps, thereby physically distinguishing them from proliferative, acetylation-dominant cell subpopulations in the oxygen-rich perivascular zone. Recent studies in non-small cell lung cancer, integrating spatial multi-omics technologies, revealed how lactate spatial heterogeneity drives local reprogramming of the immune-vascular network (196). Applying this cutting-edge multi-omics paradigm to CSC research is crucial for validating the ATME-CSCs axis: it will fundamentally demonstrate how metabolic waste from the tumor bulk (lactic acid) is spatially confined within specific hypoxic microenvironments, acting as a physicochemical signal to precisely and directionally induce a recalcitrant, therapeutically resistant stem cell subpopulation.

Furthermore, investigating whether CSCs in acidic niches remotely educate stromal cells via specific exosomal cargos remains a critical frontier (197). However, this “education” is not merely unidirectional signaling but triggers profound metabolic interactions, most notably manifested as the “reverse Warburg effect” and “stromal parasitism” (198).

An ideal “ATME-CSCs axis” model must encompass the highly complementary metabolic crosstalk between CSCs and reprogrammed stromal cells, particularly cancer-associated fibroblasts (CAFs). Recent studies integrating spatial multi-omics with single-cell mass spectrometry imaging reveal that CAFs undergo significant metabolic reprogramming within the tumor microenvironment. Their aerobic oxidation is suppressed while glycolytic pathways are strongly activated, leading to massive lactate efflux into the microenvironment. CAFs thus serve as critical “lactate donors” (199). Meanwhile, highly metabolically plastic CSCs act as “metabolic parasites.” As the oxidative population within tumors, CSCs actively uptake lactate continuously supplied by CAFs via MCT1. In this process, the reprogrammed stroma essentially actively “feeds” tumor stem cells: This ingested lactate not only enters the tricarboxylic acid (TCA) cycle to provide alternative energy fuel for CSC oxidative phosphorylation (OXPHOS) during nutrient deprivation, but also directly converts to lactyl-CoA. This continuously drives histone lactylation (e.g., H3K18la) and other epigenetic programs that maintain stemness. This metabolic parasitism—where the stroma produces acid and the stem cells consume it—forms the core biochemical feedback loop of the ATME-CSCs symbiosis axis. To this end, advanced *in vivo* models—including patient-derived organoids (PDOs) cultured under tunable physicochemical conditions and intravital imaging—will be essential to dissect the real-time evolution of this ecosystem under therapeutic pressure (200).

Building upon these mechanistic insights, the focus must shift toward developing next-generation therapeutic modalities. The “three-pillar” framework (Niche Neutralization, Metabolic Dual-Targeting, Epigenetic Reprogramming) provides a rational blueprint for creating smarter, tumor-context-responsive agents. One promising avenue lies in “Sense-and-Act” nanomedicines designed to release payloads, such as LDHA siRNA or HDAC inhibitors, specifically in response to low pH or high lactate (201). Parallel efforts should prioritize dual- or multi-specific molecules that simultaneously block CSC surface markers and immune checkpoints (e.g., CD133xPD-1) to concurrently target the CSC core and its immunosuppressive shield (202). Moreover, advancing the *in vivo* delivery of CRISPR-dCas9-based epigenetic editors offers the potential to correct disease-driving modifications, such as erasing SOX2 promoter lactylation, with locus-specific precision (203).

Ultimately, the success of these interventions hinges on building robust bridges to personalized clinical translation. To overcome tumor heterogeneity, Patient-Derived Tumor Microenvironment Organoids (PDTMOs) will serve as indispensable “avatars” for high-throughput drug testing under physiologically relevant acidic conditions, effectively predicting patient-specific therapeutic vulnerabilities (204). In tandem, the development of non-invasive, axis-specific biomarkers is paramount for patient stratification. This includes not only quantifying lactate-modified histone carriers in circulating extracellular vesicles but also employing functional imaging—such as hyperpolarized ¹³C-pyruvate MRI and pH-weighted CEST MRI—to dynamically visualize glycolytic flux and acidity during therapy (205).

In conclusion, viewing cancer through the lens of the ATME-CSC axis reframes our understanding of its resilience. The path forward lies in embracing the complexity of this dynamic system. By leveraging cutting-edge technologies to dissect its mechanics and to design sophisticated, multi-pronged interventions, we can progress from the historically intractable challenge of targeting plastic, niche-protected CSCs towards a new era of ecosystem-informed precision oncology.
